# Disentangling participation in online political discussions with a collective field experiment

**DOI:** 10.1126/sciadv.ady8022

**Published:** 2025-12-10

**Authors:** Lisa Oswald, William Small Schulz, Philipp Lorenz-Spreen

**Affiliations:** ^1^ Center for Adaptive Rationality, Max Planck Institute for Human Development, Berlin, Germany.; ^2^ Social Media Lab and Cyber Policy Center, Stanford University, Stanford, USA.; ^3^ Center Synergy of Systems and Center for Scalable Data Analytics and Artificial Intelligence, Dresden University of Technology, Dresden, Germany.

## Abstract

Online political discussions are often dominated by a small group of active users, while most remain silent. This visibility gap can distort perceptions of public opinion and fuel polarization. Using a collective field experiment on Reddit, we examined factors predicting self-selection into silent “lurker” and active “power-user” roles and tested whether participation differentials can be reduced with norm- or incentive-based interventions. We recruited 520 United States participants, randomly assigned them to conditions in six private communities, and asked them to discuss 20 political issues over 4 weeks while completing weekly surveys. Lurking (posting nothing) was most common among users who perceived discussions as toxic, disrespectful, or unconstructive; these same perceptions also predicted power usership (more posting, conditional on not lurking). Experimentally, financial incentives for commenting reduced participation differentials, whereas we did not find effects from a civility norm treatment. These findings support preference- and incentive-based accounts of participation but suggest that light-touch interventions are unlikely to bridge participation gaps, let alone polarization.

## Teaser

A field experiment examines patterns of online political discussion participation and the challenges of addressing inequality.

## INTRODUCTION

Although user-generated content is a central feature of social media, there is a stark and consequential divide between consumers and producers of content online. This divide is especially pronounced when the content is political: Most users passively view content, while a small, active minority generates most of the posts and comments. Early research on online communities coined the terms “lurkers” (*1*, *2*) and “power users” (*3*) to describe these groups. More recent studies have highlighted a particular concern: The most active content producers have been found to hold more extreme opinions than the general population, which threatens to distort citizens’ perceptions of public opinion and the norms governing public discourse (*4*–*8*). A related line of research indicates that excess toxicity in online discussions may stem from this same self-selection phenomenon (*9*–*11*), such that users perceive toxicity as more prevalent than it truly is in the underlying population (*12*).

The concern that public discourse might be distorted by differential participation echoes the so-called “spiral of silence,” theorized by Noelle-Neumann (*13*, *14*), where citizens abstain from voicing (what they believe to be) unpopular opinions. Noelle-Neumann conceptualized opinion popularity in the literal sense of majority and minority opinions, but it is evident how online spaces’ attention economies could privilege content with certain attention-grabbing tonal attributes like extremity and toxicity, making it similarly “popular” and thereby incentivizing its production. When users vary in their inclination to produce such content, we may see a “spiral of toxicity” in which hostile users produce more such content while moderate speakers lurk in the shadows. Kuran (*15*) theorizes a general utility function for political speakers, composed of their material interests in the issue at stake, the reputational consequences of voicing a given view publicly, and the speaker’s intrinsic need to voice their authentic views (and feel a sense of integrity from doing so). If the costs and benefits of online speech are unequally distributed vis-à-vis users and their political dispositions, this would be expected to give rise to unrepresentative political discourse online.

Unexpectedly, little is known about why some users are lurkers and others are power users in online political discourse (*16*), but an important prerequisite for either membership is political interest (*17*, *18*). This may be obvious for political power users, but it is equally true for lurkers, who must have sufficient interest to consume political content (otherwise, they would simply be absent from online political discourse, not lurking). Among politically interested users, however, it is not clear what set of factors is most important for determining whether a user lurks or becomes a visible user, and recent research suggests that users’ motives can be manifold (*19*). For this reason, the present study applies a longitudinal design that samples politically interested users and recruits them into online communities afresh, to prospectively observe what roles they take on within that community, and how these roles are predicted by various traits, attitudes, perceptions, and environmental factors. This addresses our first research question:

1) How are active users different from silent users—what factors predict participation in online political discussions?

The present study also tests experimental interventions designed to affect participation, using both contemporary mainstream community-moderation tools, and time-tested behavioral science methods for probing incentive structures.

Whenever we use the term “participation inequality,” we use it descriptively to denote variation in individuals’ levels of engagement in online political discussions (therefore, used interchangeably with participation gap or differential). We emphasize that our focus is primarily on behavioral differentials rather than structural or socioeconomic inequalities that constrain participation (*20*, *21*). The question of where on a spectrum between independent individual behavioral decisions and downstream consequences of structural factors online political commenting can be situated is an empirical question this study speaks to.

A common approach to improving the tone of political interactions is to raise the anticipated costs of creating toxic content, by means of rules and restrictions. For example, emphasizing group rules and norms increased the participation of newcomers in a field experiment (*22*) and using automated counterspeech to confront online hate speech succeeded in reducing racist posts (*23*, *24*). Alternatively, we can also consider interventions that aim to motivate low-output users to contribute more. Positive feedback on social media has been shown to incentivize the creation of uncivil content (*25*, *26*), and prior studies have tested the possibility of creating prosocial effects through positive feedback, such as upvotes on Reddit (*27*), awarding contributions to Wikipedia (*28*), highlighting quality content (*29*), or a “thank you” button (*30*), with positive downstream consequences for participation and content creation. We therefore aim to test examples of these two main types of intervention against online participation differentials: preventing uncivil contributions by the active minority and incentivizing the participation of the silent majority. This corresponds to our second research question:

2) Can monetary incentives or norm-based moderation reduce participation differentials, and what are the consequences for discourse and attitudes?

These two research questions have been rephrased and shortened for readability; see Materials and Methods for the full list. Our original preregistration is available online at https://doi.org/10.17605/OSF.IO/M8G4X, and all exploratory analyses are labeled as such.

### Design overview

**Fig. 1.**
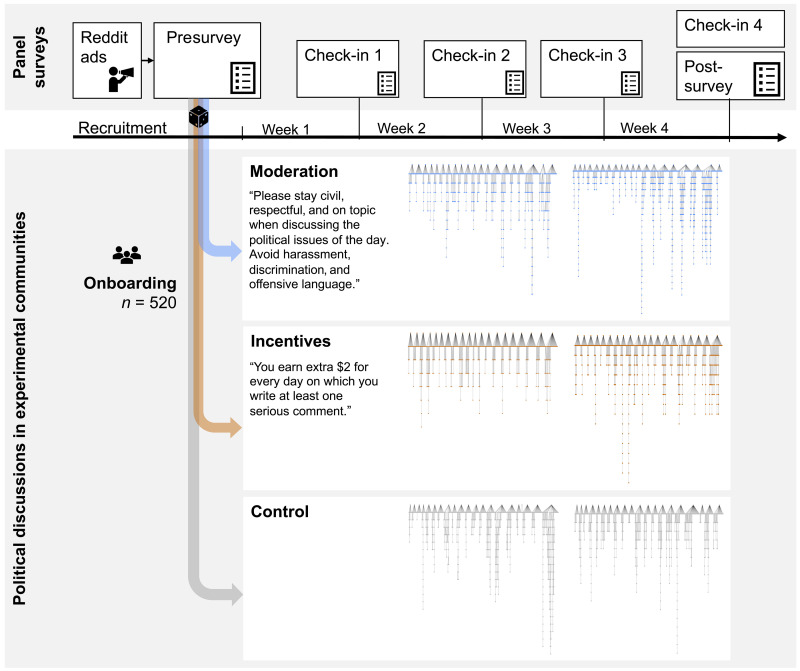
Study design including panel survey and discussion component. The study includes a 4-week discussion phase (with a check-in survey at the end of each week), bookended by a prediscussion survey and a postdiscussion survey. Six online communities on Reddit interacted under three experimental conditions (moderation, incentives, and control). Five different discussion issues were introduced every week to initiate discussions (order randomized between communities). Discussion tree networks show data for each of the six communities: The top row of nodes represents the 20 discussion seed posts by researchers; the cascades below represent comments written by participants (total participant comments within the experiment, *n* = 5819).

To address these questions, we created six large experimental communities on the popular social media platform Reddit, leveraging the site’s structure of subreddits. These are sections of Reddit managed and moderated by volunteers, typically centered around specific topics or interests. We created and maintained parallel subreddits solely for the purposes of the field experiment. Participants were recruited via Reddit Ads, which referred interested users to a baseline survey without manipulations to group composition along political ideology or attitudes. After screening out low-quality responses (see section S4 on participant onboarding), we randomly assigned participants to one of our six subreddits: two incentivized subreddits, two moderated subreddits, and two control subreddits (see Fig. 1). The experimental interventions were designed to reduce participation differentials by (i) offering monetary incentives to encourage the silent majority to become active and (ii) emphasizing civility norms to reduce discourse toxicity, thus lowering potential barriers to participation related to concerns about harassment.

In the incentive condition, participants were offered $2 for every day on which they wrote at least one serious comment, potentially adding up to $40 if they commented on each of the discussion seed posts initiated by researchers. This intervention was intended to offer an extrinsic motivation to offset the perceived barriers that, otherwise, may discourage lurkers from commenting. In the moderation condition, participants were reminded to “stay civil, respectful, and on topic when discussing the political issues of the day” and to “avoid harassment, discrimination, or offensive language.” The built-in spam filter and community control measure within Reddit’s moderator settings were additionally applied in the moderation condition. Both other conditions operated under default settings (see Fig. 1 and section S2 for details).

This design with large groups (87 members per group, on average) who were able to interact in real time allowed us to (i) study online participation in a controlled setting, without users having prior social relationships or roles in the community; (ii) randomly allocate participants to experimental groups, with treatments implemented at the group level; (iii) collect rich survey data at several points of time; (iv) analyze complete conversations and interaction networks; and (v) collect external social media data under informed consent, applying stringent ethical standards to the study of Reddit populations (*31*). Last, (vi) we were able to investigate not only the behavior of active users but also the characteristics, perceptions, and motives of those who remain silent. We are thus able to shed light on several dimensions of online participation that have previously been difficult to assess.

Our design deliberately treated entire communities, assigning participants to private subreddits that were isolated across conditions. This approach helps prevent cross-condition interference but introduces dependence within each community. However, this reflects a core feature of real-world online platforms: Interventions are experienced collectively, and their effects emerge through social interaction. We view this trade-off as necessary to capture the socially embedded nature of online engagement and believe that it provides a useful complement to more individually isolated experimental designs.

An important characteristic of interpersonal political interactions is the opinion composition of a group, as it likely affects participation, discourse, and attitude change (*32*). Discussions in this study are characterized by low heterogeneity of opinions (see figs. S4B and S18), resembling the most frequent experience on social media, which is theoretically thought to increase polarization (*33*). However, previous literature derives conflicting predictions for participation in online political discussions among largely like-minded individuals: increased genuine opinion expression (*32*, *34*) or increased conformity (*35*).

## RESULTS

The experiment took place during a 4-week discussion phase running from 10 June to 5 July 2024. It involved 520 consenting participants, who were admitted to one of six private Reddit communities after completing the onboarding (prediscussion) survey. Of these, 331 participants actively engaged during the discussion phase by posting 5819 comments in response to 120 discussion seed posts covering 20 political issues. An additional 62,981 Reddit comments made by our participants in other communities were gathered over a 12-week window framing the study. After the discussion phase, a posttreatment survey repeated the items from the prediscussion survey. Furthermore, weekly check-in surveys assessed participants’ perceptions of the discussions, the group dynamics, and the issues discussed each week. Further details can be found in Materials and Methods.

### Predictors of participation

**Fig. 2.**
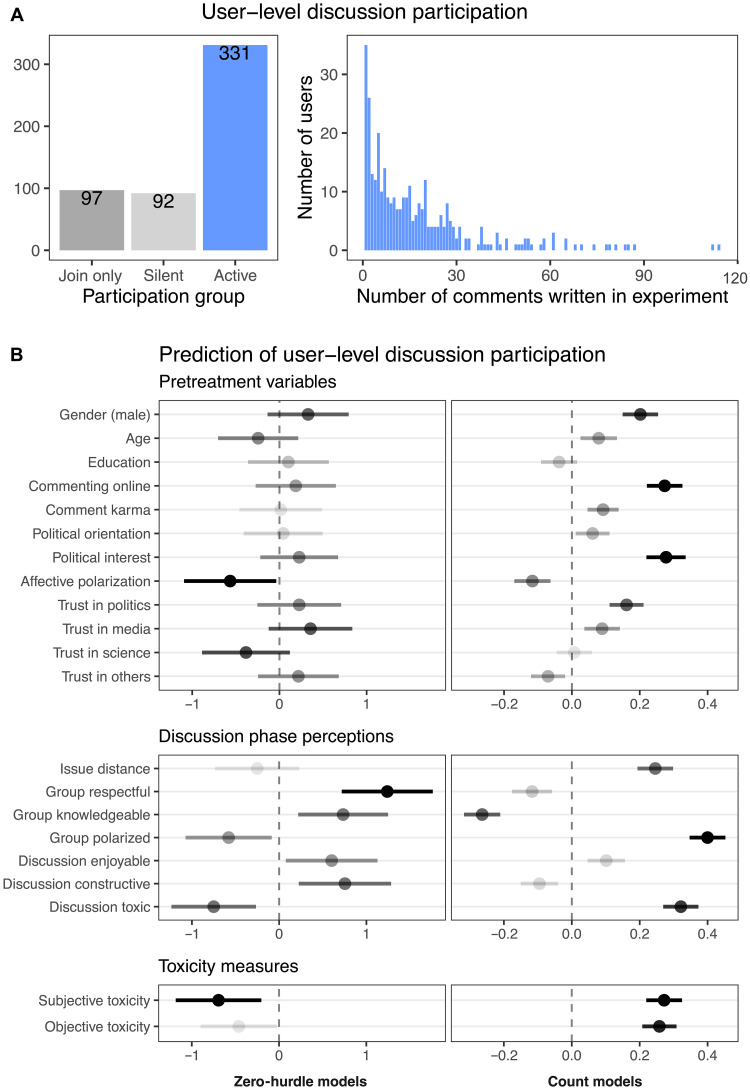
Silent versus active users. (**A**) Participant-level participation in online political discussions. Join-only participants completed the prediscussion survey and were admitted to the subreddits but did not participate further in the study and are excluded from the analysis. (**B**) Predicting the number of comments written during 4-week discussion phase (count model) and activity status (active versus passive) excluding join-only users (zero-hurdle model). Independent models estimated for each predictor variable. Predictors in upper block were measured in pretreatment survey, and predictors in the second block were measured during discussion phase in check-in surveys. Objective toxicity = average group toxicity measured using Perspective API on comments; subjective toxicity = toxicity perception in check-in survey residualized by objective toxicity. See figs. S1 and S7 for alternative model specifications and robustness.

To determine what factors are associated with discussion participation, we operationalize “lurking” as the binary behavior of writing zero comments throughout the study (versus writing one or more), and we operationalize “power usership” as the scalar number of comments written by a user during the study (among nonlurkers). Participants who neither wrote any comments nor responded to any of the check-in surveys were categorized as “join-only” cases (Fig. 2A) and were excluded from the predictive analyses on the basis that they were inattentive to the study, rather than lurkers.

This structure reflects a common challenge in modeling participation data: Many users never contribute at all, while those who do vary substantially in how much they contribute. Zero-hurdle models are designed precisely for this setting (*36*). The first component estimates the predictors of whether someone crosses the “participation hurdle” (lurker versus nonlurker), while the second component estimates the predictors of the amount of participation among those who do participate. This allows us to separately examine determinants of initial participation and the determinants of intensity of participation, rather than conflating them.

Hurdle models have been widely applied in fields where participation and intensity are theorized to arise from distinct processes, for example, to study populist alternative news use (*37*) and different forms of social media engagement (*19*, *38*, *39*). By applying the same logic to online commenting behavior, we are able to align our statistical model with the theoretical distinction between lurking versus commenting, and between light versus heavy participation.

Accordingly, we applied a two-component zero-hurdle model with a hurdle component (binomial with logit link) that models the zero counts (lurkers) and a truncated count component (Poisson with log link) that models comment counts (degree of power usership), implemented using the pscl::hurdle() R function (*36*). We display results for the full sample in the Results section, and report the breakdown by experimental group in the Supplementary Materials (see fig. S8).

We start by considering the predictors of whether a user lurks, as opposed to writing at least one comment. Our analysis using the binomial hurdle component of our model indicated no significant demographic predictors but did find significant evidence that lurkers were more likely to perceive the group as more polarized and toxic and less respectful and knowledgeable and to experience the discussions as less enjoyable and constructive, compared to nonlurkers (see Fig. 2B, zero-hurdle model). For nonlurkers, comment counts (our metric of power usership) were positively predicted by male gender, self-reported tendency to comment online, and political interest (see Fig. 2B, count model). When it comes to perceptual factors predicting comment counts among nonlurkers, what we found was unexpectedly similar to the predictors of lurking: The three strongest predictors for writing more comments (among those who wrote at least one) were perceiving the group as polarized, the discussions as toxic, and the group as not knowledgeable. Furthermore, the perceived distance between group and personal issue attitudes positively predicts the number of comments created by nonlurkers. Our results were robust to different model specifications (see figs. S1 and S7).

In our analysis of predictors of participation, we distinguish between pretreatment variables (e.g., gender and political interest), subjective perceptions (e.g., perceived discussion toxicity or perceived group characteristics) formed during the discussion phase and text-based measures of toxicity (see Fig. 2B). Given our research design, these perception reports must be understood as a mixture of “objective” variation (in, e.g., toxicity) across the six subreddits (attributable either to the experimental treatments or to emergent group dynamics), along with “subjective” variation in the users’ tendency to perceive toxicity (in a sense, their toxicity-sensitivity). To better isolate the subjective component (the user’s sensitivity to toxicity) from the objective component of perceptions (the actual toxicity of the subreddit), we residualize perceived toxicity by an objective measure of toxicity. We used the Jigsaw Perspective API to estimate the mean toxicity of participant-generated content in each community as an “objective” measure of toxicity. Measured group toxicity (aggregated over the entire discussion phase) correlated with individually perceived group toxicity (aggregated over all four check-in surveys), at correlation coefficient *r* = 0.15. Results in Fig. 2B indicated that the residualized perceptions (that is, the “purely subjective” toxicity-sensitivity component) remain predictive of participation even after adjusting for actual community-level toxicity. Furthermore, multilevel reestimation with community random effects confirms that perceptual predictors retain significance despite community-level clustering (see fig. S1), suggesting that individual subjective evaluations, regardless of origin, are central to understanding participation. Additionally, when we substitute the objective toxicity metric for the perception reports in the main analysis (completely eliminating the subjective component), the original association holds. This indicates that both objective toxicity and subjective toxicity-sensitivity matter for self-selection. In summary, the objective toxicity of the discussion groups interacted with users’ own sensitivities to toxicity, catalyzing participation gaps by exerting opposing self-selection effects for lurkers and power users.

### Incentives and moderation treatments

#### Did the treatments affect participation?

**Fig. 3.**
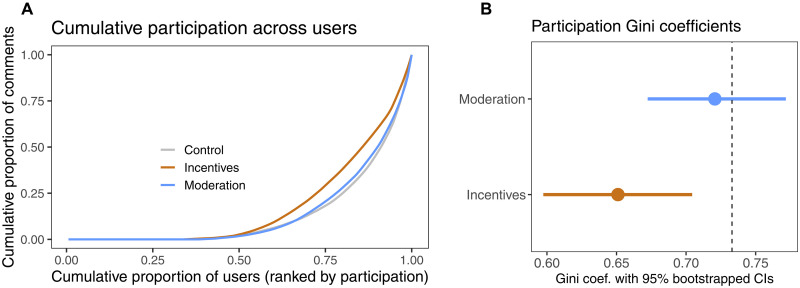
Treatment effects on participation. (**A**) Cumulative participation distributions by experimental condition. (A diagonal line would suggest equal participation across users.) (**B**) Gini coefficients of participation inequality with bootstrapped 95% confidence intervals (CIs). The dashed line represents the control group’s Gini coefficient.

We next examined whether our experimental interventions—namely, incentives for commenting and moderation norms—succeeded in reducing participation differentials. Figure 3A presents participation distributions by experimental condition, and Fig. 3B plots Gini coefficients as indicators of participation inequality. As expected, the incentive intervention led to a significant decrease in participation inequality. However, the effect was limited when considering the strong incentive ($2 for every day of commenting). The moderation treatment did not reduce participation inequality.

**Fig. 4.**
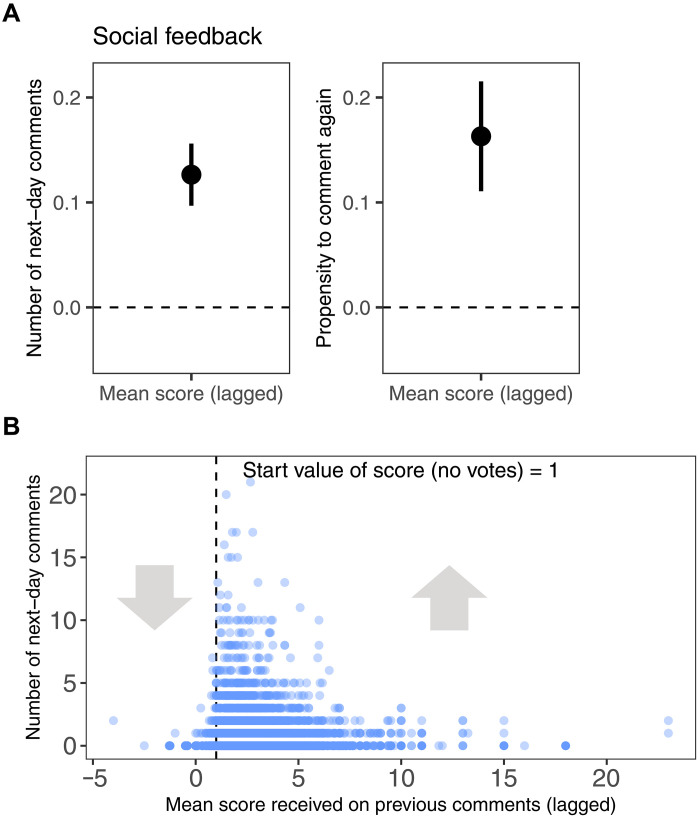
Social feedback (voting) effects on subsequent comment activity. (**A**) Receiving positive votes on comments was associated with increased subsequent commenting (left: Poisson generalized linear mixed-effects model predicting the number of next-day comments; right: binomial generalized linear mixed-effects model predicting the propensity to comment again on the next day, partial pooling applied in both models). (**B**) Lagged mean scores received for previous comments and number of subsequent comments, with score = 1 as default if no voting occurs. Values above 1 indicate net positive feedback; values below 1 indicate net negative feedback.

While the treatment effects represent differences between communities, to further investigate path dependencies within our communities with regard to participation, we conducted an exploratory test to determine the relationship between commenting activity and social feedback received on prior comments. More specifically, we investigated whether the number of comments made by a participant on a particular day of the study was predicted by the mean score (in terms of the balance of upvotes-downvotes) of all the comments that they had written on prior days of the study. We found a significant positive effect of social feedback: Receiving positive votes on comments was associated with increased subsequent commenting (see Fig. 4A). This effect was reflected in a discontinuity at the cutoff score of 1, which represents Reddit’s start value if no upvoting or downvoting occurs. A score greater than 1 implies net positive social feedback (more upvotes than downvotes), whereas a score below 1 indicates negative social feedback (see Fig. 4B). See section S6.3 for more details on within-group dynamics.

#### Did the treatments reduce discourse toxicity?

**Fig. 5.**
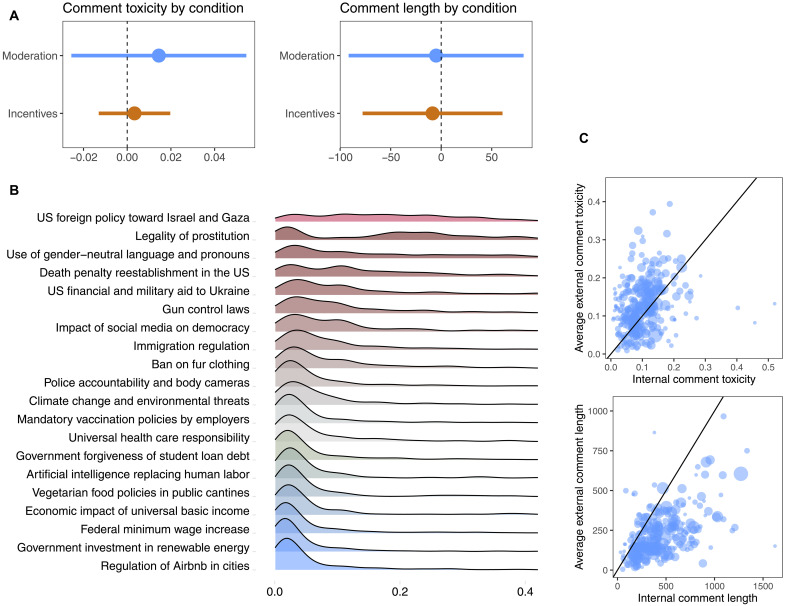
Treatment effects on discourse. (**A**) Treatment effects on comment toxicity (left) and comment length (right). Linear mixed models estimated at the comment level with subreddit level random effects; treatment conditions are compared to the control condition. (**B**) Toxicity distributions for 20 discussion issues (Jigsaw Perspective API, scale of 0 to 1). (**C**) Observer effects on comment characteristics. Top: Internal versus external comment toxicity. Bottom: Internal versus external comment length, diagonal highlighted (external Reddit activity collected under informed consent.)

Considering the downstream consequences of the two experimental treatments for discourse quality, we examine changes in comment toxicity. Given the subreddit-level dependencies within the structure of our experimental design, participants interacting in groups, we implement a random-effects specification that allows us to acknowledge subreddit-level dependence while still making use of the large number of individual observations, using an approach that reflects the data-generating process, in which both treatment and subreddit context shape responses (see section S6.4 for a discussion on statistical power). We found that, contrary to expectations, neither moderation nor incentivization reduced comment toxicity (see Fig. 5A, left). Note, however, that comment toxicity was very low throughout the study and across almost all political issues (mean of 0.11 and *SD* of 0.13, on a scale ranging from 0 to 1; see Fig. 5B).

#### Did the treatments change attitudes?

**Fig. 6.**
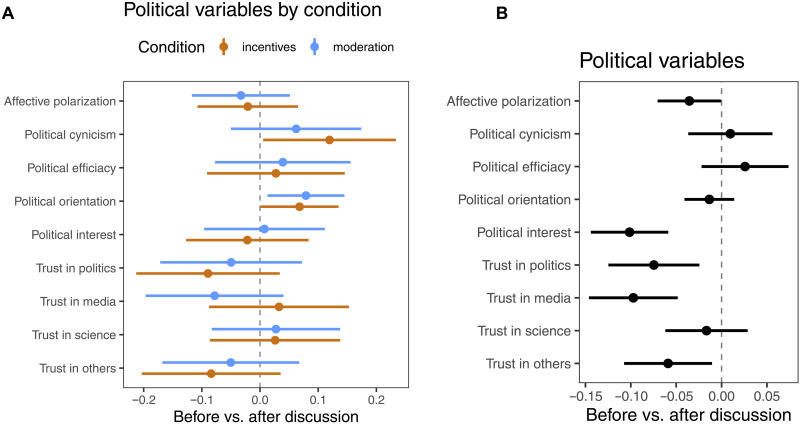
Opinion dynamics over time: Individual presurvey versus postsurvey values. (**A**) Changes in political variables by condition. Coefficients represent interaction terms between time (postdiscussion) and condition (control as reference category) for the respective outcomes. Independent linear mixed models with participant random intercepts and subreddit random slopes. (**B**) Changes in political variables across the entire sample (conditions pooled); independent linear mixed models with participant and community random intercepts.

While our treatments were designed to influence participation and further downstream discourse quality, we also collected pre- and postsurvey measures of political attitudes, including affective polarization, political trust, and perceived knowledge. Drawing again on a mixed-model specification with random effects on the level of the subreddits, we find no significant differences in changes of these outcomes between treatment groups and the control condition (Fig. 6A). Likewise, we did not find any notable differences between active and silent users in terms of their political opinion formation over the course of the experiment (see fig. S11). Given that the interventions did not directly target political cognition and that observed effects on participation and discourse were limited, these null findings are consistent with expectations. We note that even statistically well-powered interventions directly tailored toward specific outcomes often struggle to shift political attitudes.

Moving beyond the core experimental treatments and preregistered research questions, we descriptively explored opinion dynamics over time in the full sample (conditions pooled). Using linear mixed-effects models that account for nesting within participants and discussion groups with random intercepts (Fig. 6B), we found several average changes over the 4-week discussion phase. Averaged across conditions, participants’ positioning on the left-right scale tended to shift leftward (Fig. 6B), aligning with the majority political orientation of the sample. Additionally, we observed a reduction in affective polarization. However, we also found lower levels of trust in politics, media, and others in general, as well as a reduction in self-reported political interest. In line with lower reported political interest after the 4-week discussion phase, we observed consistently lower levels of reported issue knowledge for almost all of the 20 political issues discussed (see fig. S3). It remains important to note that these time effects are subject to considerable confounding by external political events.

## DISCUSSION

Online political discussions are characterized by extremely unequal participation: A minority of users contribute to the discussion, but most remain silent observers. In our lab-in-the-field setting, we found that this pattern largely persisted, even under the experimental treatment conditions designed to mitigate it. Our results thus attest to the stability of participation patterns found in previous research. Following the empirical nature of our study, the observed participation gap may reflect behavioral choices rather than structural forces that are traditionally attached to the term “inequality” (*20*). While we make no normative claims about ideal participation levels, recognizing these behavioral differentials helps clarify how online discourse amplifies a small subset of highly active voices. The unique design of our study provides insights into the possible origins of these patterns, several of which do hint toward structural path dependencies consistent with existing social science research on participation.

The finding that male participants tend to comment significantly more often than female or nonbinary participants mirrors previous findings from studies on political deliberation (*40*). Likewise, the finding that political interest is a positive predictor of online political engagement closely aligns with the literature (*9*, *41*, *42*). Even the finding that community-level toxicity predicts more commenting among active users, a result that might seem counterintuitive, aligns with recent research, suggesting that toxicity does not necessarily discourage participation and may even motivate a minority of users to be particularly active, thereby stimulating discussions (*43*). Previous research has observed how certain forms of incivility can stimulate discussions among like-minded people (*44*, *45*), perhaps partly by being entertaining (*46*) or because they represent acts of controversial political dissent (*47*–*49*). These observations may help make sense of the finding that the perceived distance between group and personal attitudes to political issues positively predicts the number of comments created by active users (in the count model), which would on average be inconsistent with predictions of the original spiral of silence theory (*13*, *14*). However, our finding aligns with previous evidence, suggesting that people with strong opinion opposition are more likely to voice this compared to people with more moderate opinions (*50*), which aligns with predictions of a mechanism in which voices of a moderate majority remain silent (*32*), although we note that, in this discussion context, the distribution of self-placed political orientation is highly left skewed, with high-attitude congruence across the largely liberal sample (see figs. S4B and S18). Last, considering discussion quality, results from the zero model closely align with the silencing effect of peer toxicity found in other recent work describing political comment sections on YouTube (*51*).

Our research design also offers a rare opportunity to observe a cohort of members in a new online community, including those who go on to become lurkers (typically excluded from the sampling frame of analyses based on digital trace data) and those who go on to become power users. The factors that predict whether users write any comments at all are almost a mirror image of the factors that predict how many comments are made by the self-selected subset of users who pass this zero hurdle. Participants were more likely to step out of the shadows and make their first post if they perceived the community as respectful, knowledgeable, nonpolarized, enjoyable, constructive, and nontoxic—intuitive criteria for deciding whether to speak up in any community. However, for users who were already active, the same variables (except enjoyment) were negatively associated with the number of comments posted.

Although these findings may initially appear contradictory, they are consistent with the sequential logic of the selection process observed: In the first stage, users are more likely to pass the zero hurdle and start posting (that is, stop being lurkers) in communities that they perceive to be respectful and constructive. Yet this intuitive self-selection pattern paradoxically results in a cohort where the power users are those who may be less sensitive to toxicity. Our decomposition of toxicity perceptions into objective (classifier-measured toxicity of the six subreddits) and subjective (residual user-level sensitivity to toxicity) components confirmed that both contributed to the selection process: Objective toxicity suppressed participation (although it should be noted that this was not attributable to the experimental treatments, which did not substantially affect toxicity at the subreddit level), and within communities of (by construction) fixed toxicity, individual users were more likely to participate the less toxicity sensitive they were, creating an active-user population that is perhaps particularly disposed to what prior research has termed “status-seeking risk-taking” (*10*).

Our experimental results validate the conceptual approach of considering the interaction of users’ dispositions and their incentives, in that financial compensation significantly equalized participation. However, this effect was limited, especially given the strength of the treatment, which we interpret as a sign of the robustness of the self-selection mechanisms at play in the phenomenon that we are studying, the subversion of which is no trivial task.

One important conclusion from modeling the treatment effects on toxicity and observing substantially larger SEs in the moderation condition compared to the incentive condition is that online communities that appear to be similar on the surface can quickly develop divergent patterns of participation and discourse. Our exploratory analyses of social feedback suggest that commenting behavior may be shaped by user popularity, which has previously been shown to be highly sensitive to random differences in initial popularity. Future research should consider how to isolate the effects of community-level treatments from the dependency structures that characterize popularity and other path-dependent processes in online communities.

To contextualize these findings, however, it is important to note that comment toxicity was very low throughout the study, leaving little room for the civility norm messages in the moderation condition to effect the intended improvement. Furthermore, the alignment between personal and perceived group attitudes to political issues was high, with the sample showing a liberal skew. The variables that predicted higher comment counts in the incentive condition echo those that predicted overcoming the zero hurdle in the full sample, namely, perceptions of the environment as respectful and constructive. The precise mechanism behind this pattern is unclear, but it may suggest ways of lowering barriers to active participation in public discourse.

Regarding the observed decline in self-reported political interest, it is worth noting that comments posted in the study were considerably longer than participants’ other Reddit comments (Fig. 5C, bottom). Several participants explicitly indicated that they “could not add anything”; some even felt “intimidated by the sophistication” of the discussions, suggesting that participants may have answered the second question on political interest more conservatively due to upward social comparison with other participants. Furthermore, the corresponding reduction in self-reported issue knowledge may reflect patterns of increased epistemic humility, a factor likely beneficial for constructive political discussions (*52*). Future research should consider including a no-discussion pure control group, to probe these findings further and mitigate temporal confounds.

### Implications

The effects of our experimental interventions were limited, which may chasten hopes for low-profile interventions to equalize online discourse participation. Our findings point to potential benefits of providing positive reasons for posting, because offering external incentives for participation somewhat equalized participation. While incentivizing participants to write comments may seem unrealistic, it could be implemented through other forms of rewards (e.g., social or symbolic) on social media platforms. Additionally, imposing caps on participation (e.g., limiting the number of comments per user per hour) could indirectly encourage a more diverse set of voices by curbing the dominance of a highly active minority.

Meanwhile, while also considering limitations in statistical power, emphasizing moderation did not further reduce the already low level of comment toxicity. However, rather than dismissing content moderation or the creation of community rules based on our findings, we draw attention to the specific nature of our treatment, which was primarily reminding participants to stay civil. If these norms had been not only declared but also enforced (e.g., by removing or penalizing toxic comments), then we might have observed the intended reduction in subsequent toxic behaviors. When considering the interplay between toxicity and participation, deplatforming central spreaders of toxicity appears promising in light of our findings as well as previous work (*53*). Future studies should consider the effects of enforcing moderation decisions on the user and community levels while carefully considering challenges to achieve sufficient statistical power with dependencies on the level of communities, potentially using our data as basis for a priori estimations of statistical power.

Our finding that comment counts (our metric of power usership) are predicted by stable personal characteristics such as male gender and a high level of political interest implies that light-touch interventions are unlikely to change who drives online political discussions. However, we also found that, for those at the threshold between silent observation and writing a first comment, perceptions of the environment are highly relevant: Both the objective toxicity of the community and the subjective sensitivities of the users interact to produce a perverse self-selection pattern that is difficult to counteract. However, to the extent that moderation with enforcement can change at least the visible toxicity of an environment, the encouraging effect this could have on would-be lurkers might make the community less toxic in reality over time.

From a methodological perspective, complex patterns such as these illustrate the importance of user-centric data collection (*54*), which starts by recruiting and surveying participants and only subsequently collects digital trace data to bridge the evidence gap between those whose behavior is active and visible and those who remain silent and, therefore, largely invisible in platform data. The method used here demonstrates that collective experiments can be independently conducted in the field on existing social media platforms. In the case of Reddit, we were able to leverage the subreddit structure to separate participant groups, which is essential for collective experiments. What is more, we were able to do this without directly collaborating with the platform, thereby avoiding potential conflicts of interest. While this approach may not be feasible on all platforms, there are various ways to implement group-level treatments and interactions in future research (e.g., with WhatsApp groups or interactive online experiments). Such experiments are crucial for advancing the understanding of collective dynamics on social media, which affect politics worldwide and are necessary given the current lack of access to large-scale A/B testing by corporations.

Presuming even considerably higher research resources (at a scale that now only social media companies hold), future researchers could build on our design by increasing the number of communities created (increasing the sample size of communities over which the treatment effects are marginalized), accumulating observational knowledge about which community features are most consequential for certain treatments, and even deliberately designing studies that construct contrasting community compositions to explore how they may respond differently to the same intervention. While this certainly is an analytically complex research setting, we believe that it is necessary to generate practical research findings that can be translated to real-world platforms where treatment effects will, inevitably, be shaped by the composition of the active user population, the predictors of which are a core object of our present analyses.

In sum, our method enabled us to measure group-level outcomes in a semicontrolled setting with high ecological validity that is typically only available to platform owners. We hope that this approach inspires future experiments on collective online behavior. Our results show that the global pattern of online participation gaps is stable, but that characteristics of the discussion climate shape participation differently for different user groups: The active minority appears motivated by toxic debates, while the largely silent majority is deterred by them. This dynamic is concerning because only the active minority is visible online, shaping perceptions of public opinion for all. Our experimental observations from incentivized participants, coupled with our observational findings about self-selection and positive social feedback, suggest that future interventions may be able to make online political discussions more representative by offering more positive social rewards for lurkers to post and by reducing the perceptible toxicity of the environment.

## MATERIALS AND METHODS

The study was approved by the Internal Ethics Board of the Max Planck Institute for Human Development, Berlin, on 5 February 2024.

### Sample

The onboarding prediscussion survey was completed by 788 Reddit users. Of these, 520 were admitted to one of six Reddit communities after we had confirmed the validity of their Reddit accounts and survey responses (for a summary of demographic characteristics of the sample, see fig. S4). The attrition rate during the admission process was thus 34% (29% in the control condition, 37% in the incentive condition, and 36% in the moderation condition). Of those admitted, 369 participants completed the posttreatment survey and at least one check-in survey, resulting in a total postadmission attrition rate of 29%, with no significant differences over the 4-week period between conditions: 28% in the control condition, 31% in the incentive condition, and 28% in the moderation condition. Exploring individual-level predictors of attrition, more specifically predictive factors for the join-only status of respondent (not following-up with researchers and not writing any comments after being admitted to the subreddits) as opposed to lurkers or active users, we find that only two pretreatment variables show statistically significant negative association with the postadmission attrition (or study noncompliance): right-wing political orientation and the self-reported tendency to often write comments online. We report results of a binomial model in the Supplementary Materials (see table S1). There is no such association for the treatment indicator as well as the covariates: age, gender, education, political interest, affective polarization, or comment karma (a Reddit internal metric of past engagement). See also fig. S2 for survival analyses of discussion dropout.

Our sample of Reddit users includes 251 participants identifying as male, 239 as female, and 30 as nonbinary. Participants span a wide age range (mean of 43.42 years and SD of 13.75) and report diverse educational backgrounds, although skewed toward higher educational attainment. A majority hold at least a bachelor’s degree, with a substantial shareholding postgraduate qualifications. Most participants report spending notable time online and on social media platforms each day, with many accessing social media multiple times per day or more. Politically, the sample is skewed toward the liberal end of the spectrum, with most participants placing themselves left of center. Political interest is high overall: More than half of the sample report being “very interested” in politics. Consistent with the ideological profile, average affective polarization is pronounced: Participants tend to rate the opposing party substantially less favorably than their own. The sample is attitudinally relatively homogeneous, but several issues (including immigration, vaccination, gender, housing, and the regulation of social media) show rather uniform opinion distributions. Additional distributions of demographic and attitudinal variables are provided in the Supplementary Materials. In line with this important observation, we would like to highlight that understanding dynamics within affectively polarized (or, at least, attitudinally homogeneous) groups is important, aligning with our emphasis on external validity in our experimental design, as these groups make up the majority of interactions in online environments.

Whether our findings would generalize to other major platforms, like Facebook, X, Instagram, or BlueSky, which have different features and communities, or to future platforms whose user communities do not yet exist is an open empirical question. Pragmatically, studies like ours are constrained to inspecting platforms that are open to data collection, and Reddit is the most popular platform that is sufficiently open to make our research design feasible. Besides the question to what extent our findings may generalize to political discussions on other platforms, it is important to note that this is a US-based study and that it is very plausible that the cultural context matters for dynamics of discussion participation. Furthermore, we are specifically considering political discussions, which come with certain specifics that we discuss above. Therefore, our findings may not generalize to discussions in other contexts, for example, the workplace or leisure activities (e.g., discussing movies, etc.).

### Discussion data

Survey responses were collected in five waves (prediscussion survey, postdiscussion survey, and four check-in surveys; see Fig. 1). Further, we collected *n* = 5819 comments that participants wrote in response to 120 discussion seed posts initiated by researchers [20 political issues (Fig. 5C), each discussed in six communities]. Of the 520 admitted users, 331 wrote at least one comment in the experiment; the average number of comments was 17 (*SD* = 19 and *max* = 114).

In addition to discussions within the study’s communities, we also collected data on participants’ discussion behavior outside the study context with their informed consent. Over a 12-week period that included the study’s 4-week discussion phase (from 13 May 2024 to 8 August 2024), we collected 62,981 external Reddit comments from participants (toxicity, measured via Jigsaw’s Perspective API: mean of 0.14 and *SD* of 0.19).

### Study procedure

Participants located in the US were recruited via Reddit Ads and invited to take an onboarding, prediscussion survey. This began with a detailed explanation of the study procedure, featuring both written and visual information, as well as a comprehension check and informed consent. It continued with questions on demographics, online activity, and political variables. Participants were then assigned to one of the three experimental conditions (control, incentives, or moderation) and invited to join one of the six private online political discussions groups on Reddit. Compensation for participation was provided in the form of Amazon gift vouchers, both after the prediscussion survey and at the end of the study. Recruitment started on 9 May 2024; the discussion phase took place from 10 June to 5 July 2024.

Participants in the incentive condition received extra compensation for active participation, again in the form of Amazon gift vouchers. They were informed about this in the prediscussion survey (along with a comprehension check), in a welcome message after joining the subreddit, and in the first post within the subreddit. Participants in the moderation condition were notified about the community rules—to be “civil, respectful, and on-topic while discussing the political issues of the day” and to “avoid harassment, discrimination, or offensive language”—via the same channels [similar to Matias (*22*)]. Furthermore, we activated automated moderation settings built into the Reddit moderator tools (see section S2 for details on subreddit settings), making the moderation condition a combined treatment. Participants in the control condition took part without additional incentives, community rules, or changes to the platform’s internal settings. There were two parallel groups for every condition for two reasons: first, to maintain a backup in case one group had to be closed for reasons such as harassment or promotion of illegal content; second, to allow the observation of developments within and between conditions. For example, differences between groups within one condition could reveal path dependencies and self-organization principles in social networks. We prioritized group size over the number of groups, in contrast to previous studies (*39*, *55*), to create an ecologically valid setting for studying public discourse dynamics on social media.

Once the entire sample had taken the first survey and we had checked the validity of the Reddit accounts and completeness of survey information, we admitted all participants simultaneously to the groups to avoid trickle-in effects. Participants discussed 20 political issues over the course of 4 weeks. Each discussion week was followed by a check-in survey, asking about participants’ motives for commenting (fig. S3) and their perceptions of the group and its discussions that week (fig. S10). The political issues were posted in randomized order and at random times between 7:00 a.m. and 7:00 p.m. US Central Time, in the form of political statements balanced in political leaning (10 left-leaning and 10 right-leaning statements), each accompanied by a discussion prompt. The groups were controlled and monitored by the researchers. However, the researchers did not actively moderate the discussions, except for one case in which a participant was excluded for posting illegal content. The handling of such exceptional cases was outlined in the preregistration to ensure the protection of other participants. The Reddit API was used to send reminders to all participants to take the final, postdiscussion survey; all other communication with participants took place through public posts to the communities, flagged as “study information,” or, occasionally, via individual messages to clarify questions.

To test the technical infrastructure, including the Reddit Ads recruitment campaign, the surveys, and the transition to the online community, we conducted a pilot study with *n* = 50 participants in one online community discussing five political issues under the control condition over 1 week. More details on participant recruitment, onboarding, the technical setup of the subreddits, participant compensation, data collection via the Reddit API, and data management are provided in the Supplementary Materials.

### Data collection

Surveys were hosted on Qualtrics. The prediscussion survey contained questions about participants’ age, gender, education, online activity, political issue attitudes, overall political orientation, and trust. All questions except those tapping demographic information were repeated in the postdiscussion survey, which also included additional questions on participants’ reasons for not writing comments and their overall perception of the study. The weekly check-in surveys contained an exposure check asking which topics were discussed that week. It also assessed participants’ self-reported forms of participation, use of ChatGPT in the study (which was largely absent), motives for participating, and their perception of the group, the discussions, and participants’ and others’ issue attitudes (see figs. S3, S4, and S10 and tables S3 and S4). Discussion data were collected using the Reddit API under informed consent. We collected posts and comments from the experimental subreddits, including the full text, timestamps, votes, authors, and reply structure. For every user, we also collected the “comment karma,” a Reddit-specific cumulative measure of past activity on the platform, as well as a set of comments written outside the experiment. For political issue seed posts, the “post insights” metric of total views was collected manually, as there was no API endpoint at the time of the study.

### Analysis plan

The following research questions were formulated in the pre-analysis plan:

1) How do actively and passively participating users in a subreddit differ with regard to demographics, political variables, psychological variables, and other online activity?

2) How do actively and passively participating users differ with regard to their political opinion change between the pre- and postdiscussion survey?

3) To what extent is the decision to comment predicted by the perception of the group?

4) Can we manipulate activity levels of participation with experimental treatments (incentives and moderation)?

5) Does the treatment (with the intent to manipulate participation) affect discourse quality and structure?

6) Does the treatment (with the intent to manipulate participation) affect political variable changes between the pre- and postdiscussion survey?

7) Are political opinion changes mediated by discourse quality and structure?

Results addressing the first three questions are reported for the full sample due to the observational nature of RQ1. As we are not trying to estimate any causal treatment effect here, the fact that some of the variation in perceptions may have been exogenously driven by the treatment assignments is not intrinsically problematic, in particular, given the limited effects of experimental treatments. Results for the control condition separately are reported in the Supplementary Materials (see fig. S8). They mirror the results from the full sample but suffer from substantially reduced statistical power. To explore the implications of community-level clustering of observations, we reestimated our main models with community-level random effects. Results indicate increased uncertainty (wider confidence intervals) but stable patterns of predictors (see fig. S1). Results addressing downstream consequences for opinion change (questions 2 and 6; see Fig. 6) did not provide any substantial insights that would justify giving them a similar amount of space as the results on participation, which are the theoretical focus of this paper. Question 7 was omitted as there were no significant changes in opinion between conditions. We report toxicity and comment length as the primary discourse metrics, as other preregistered measures are still under development.

The following analyses were exploratory: social feedback models (Fig. 4) and pooled descriptive changes in political variables over time (Fig. 6B), as well as any supplementary analyses presented in the Supplementary Materials.

### Ecological validity

This study allows for the granular observation of political discussions in large groups under experimentally controlled conditions. Collecting data on participants’ discussion behavior outside the study made it possible to explore observer effects and other methodological artifacts from the study setup (see fig. S6). When asked explicitly about the perceived discrepancy between the study context and their regular experience on Reddit, participants indicated discussion toxicity, enjoyment, and group respectfulness to be somewhat different than usual. We also found a correspondence between the number of comments a participant wrote during the study and their (log-transformed) comment karma values, a cumulative summary metric of past comment engagement on Reddit (*r* = 0.14). Likewise, we found a correspondence between users’ average internal and external comment toxicity (*r* = 0.22) and, especially, average internal and external comment length (*r* = 0.56; Fig. 5C). Note, however, that the average comment length was systematically higher in our study than in participants’ external Reddit usage, whereas average comment toxicity was systematically lower.

Concerns regarding observer effects, such as a significant reduction in the length of comments through a potential loss of intrinsic motivation when introducing external reward (*56*), did not materialize in our design (Fig. 5A, right), and other metrics of “satisficing” behavior showed no difference between the incentivized and other conditions (see section S6.5 for a discussion on overjustification).
