# Supplementary material for: Disentangling participation in online political discussions with a collective field experiment
Source: Sci Adv. 2025 Dec 10;11(50):eady8022. doi: 10.1126/sciadv.ady8022 (PMC12693982; doi:10.1126/sciadv.ady8022)
Supplement: Supplementary file 1 — Supplementary Text Figs. S1 to S18 Tables S1 to S4 [file sciadv.ady8022_sm.pdf]

Science Advances
AAAS

Supplementary Materials for

Disentangling participation in online political discussions with a collective field experiment

Lisa Oswald *et al*.

Corresponding author: Lisa Oswald, oswald@mpib-berlin.mpg.de

Sci. Adv. **11**, eady8022 (2025)
DOI: 10.1126/sciadv.ady8022

This PDF file includes:

Supplementary Text
Figs. S1 to S18
Tables S1 to S4

1 Participant recruitment

Participants were recruited via Reddit Ads posted from an Ad account that transparently identified the advertisers as researchers from their institution (see Fig. S16). The text of the Ad was the following: “Hi Redditors, we are researchers at the [institution] in [city]. We are looking for participants for a compensated study! The study includes taking surveys and discussing political issues in a private subreddit.” The Ad was fielded to English speakers based in the United States. Our bidding strategy involved using a daily budget over a period of 28 days.

2 Details on subreddit settings

Across all subreddits, only “self posts”—that is, posts that contained text but no images or links to image hosting sites—were allowed. The community information contained a note instructing participants to avoid meta-discussions about the study.

The following text was pinned to the top of the subreddit in the onboarding phase of the experiment: “This community is all about discussing politics! (Heads up—we’re a text-only community, so no images or links please.) We are part of a research study and moderated by researchers of the [institution] in [city]—feel free to message the mods if you have any questions.”

The following messages were sent to participants after admitting them to the subreddit: “Welcome to r/[subreddit]! This community is all about discussing political issues. It is part of a research study and is moderated by researchers at the [institution] in [city]. We’re posting one new issue every weekday (Monday–Friday) for four weeks. Topics will be posted at random times between 7am and 7pm CST, so please set notifications for this community to “Frequent” so you don’t miss it when a new topic is posted! Check out the wiki and the sidebar for general information and guidelines on the study. Otherwise, feel free to message the mods if you have any questions or concerns. Heads up—we’re a text-only community, so no images, links, or cross-posting.”

In the incentives condition, the following text was added: "Remember, for every day on which you write at least one serious comment, you collect an extra $2. For example, if you comment every working day, you'll receive an extra $40 Amazon eGift Voucher at the end of the study!"

In the moderation condition, the following community rules were stated: "Please be civil, respectful, and on-topic while discussing the political issues of the day. Avoid harassment, discrimination, or offensive language. We use strict spam filters and reserve the right to selectively remove users from the community due to non-compliance with our rules. Anyone removed from the community will be excluded from further participation in the study." Furthermore, Crowd Control was set to "moderate" for posts and comments to "automatically collapse comments from users you're not sure about"; spam filter strength was set to "high" for posts and comments. Both other conditions operated under default settings.

3 Participant compensation

Participants were compensated for their participation in the study with gift vouchers. They received a $10 gift voucher after successfully completing the first survey and joining the subreddit, and a $30 gift voucher after completing all 4 check-in surveys (worth $5 each) and the final survey. Participants who missed surveys received partial compensation. Additionally, participants in the incentives condition received an extra $2 towards their second voucher for every day they wrote at least one comment.

Amazon eGift Vouchers were chosen for three reasons: they are very popular in the United States, they can be used for a wide variety of items, and the value can be chosen freely. In contrast, other options like AMEX eGiftcards have a minimum value of 25$.

4 Participant onboarding

Before participants were admitted to their assigned experimental subreddit, the survey data were carefully checked for completeness and signs of duplicate survey taking. We checked the Reddit accounts for shadow banning by Reddit and for their date of creation to mitigate fraudulent behaviour. In this field setting, we cannot entirely rule out spillover between experimental conditions because there is no definite way to determine whether each participant corresponds to a unique person who participated with no knowledge of other conditions. First, it is possible that individuals participated multiple times with distinct Reddit accounts and different email addresses. To mitigate this possibility of fraudulent participation, we did not admit participants with Reddit accounts that were created after recruitment started or that were shadow-banned by Reddit (clearly labeled as such by Reddit) or who took less than 5 minutes to complete the pre-survey (which corresponds to the lowest 1% percentile) to the subreddits. Second, to maintain random assignment, a small number of participants who took the pre-survey twice due to technical issues (e.g., inability to join private subreddits via the mobile app during the recruitment phase, requiring them to switch devices) were admitted to their originally assigned subreddit after correspondence with the researchers. Although the main treatment was administered during the discussion phase in the subreddits, participants might be aware that other experimental conditions exist. To mitigate the associated risks, participants were reminded in the subreddit sidebars to avoid meta-discussion about the study. Finally, although very unlikely, it is possible that different members of the same household participated in the experiment but under different conditions. For future studies following a similar methodology, we summarize a set of recommendations on how to minimize spillover, bots, and repeated participants. First, we recommend providing wiki-type sites to participants during onboarding and encouraging them to bookmark the site to minimize individual

communication with researchers, avoid repeated survey taking for informational purposes, and lend legitimacy to the study for more skeptical participants. Second, we recommend using unique, trackable clickthrough links for each individual, which always direct to the same condition, and implementing metadata trackers from the start of the study, which can help to identify duplicates. Third, we recommend implementing bot detection measures early within the first survey.

5 Discussion phase

The order of the 20 seed topics was randomized between subreddits. The seed posts included a title (top line of the post), which corresponded to the issue attitude statements presented in the surveys, and the following subtext: "Please discuss! This statement serves as starting point for today's discussions. It neither reflects the opinion of the researchers nor a political position of the research institution." The seed posts were scheduled for between-subreddit randomized times, in a window between 7am and 7pm US Central Time. Seeds were posted every working day (Monday to Friday) for four weeks. Links to check-in surveys were posted on Saturdays, 7am Central Time. The post of the day was highlighted by a flair tag: "Daily discussion topic."

We did not use any moderator response automation, except for an automated flag to mitigate meta-discussions in the subreddit. If participants attempted to write a post containing any of the words *study, experiment, voucher* or any of the names of other subreddits, they were shown the following message "It looks like you might be posting a meta-discussion about the study. Please avoid this, as it may jeopardize our methodology! If you have any questions, please refer to the sub's wiki or contact the mods."

The researchers monitored the unfolding discussions continuously over the four-week discussion period using a custom feed that contained the six experimental subreddits. Participant questions were mostly answered individually via Reddit mod mail, and sometimes via direct chat messages on Reddit, email, or in the form of comments to public administrative posts in the subreddits, such as reminders to take a check-in survey.

6 Supplementary Analyses

6.1 Participation prediction including random effects

We re-examined the hurdle models with a particular focus on the nested structure of the data, including community random effects. We find that a considerable share of variance occurs on the level of communities, widening error bounds around our estimates. However, the core pattern of predictors remains unchanged for the count model as well as the zero model (see Fig. S1).

6.2 Differences between treatment groups in participation hurdle models

In an exploratory analysis, we investigated whether the descriptive predictors of participation were similar across experimental conditions. We found diverging patterns between the two treatment conditions. In the moderation condition, comment counts were positively predicted by male gender, political interest, perceiving the group as *not* knowledgeable and the discussions as toxic. In the incentives condition, comment counts were predicted by political interest, perceiving the group as knowledgeable and the discussions as *not* toxic (Fig. S8). Baseline balance tests

found no indication of significant differences between the experimental groups prior to treatment (see Table S3 and S4 for baseline balance tests). It is possible that the experimental conditions happened to be imbalanced on a psychological dimension that we did not measure at baseline, and that this predicts the divergent patterns of behavior between the groups. However, it is equally plausible that discussion dynamics emerged differently over the course of the study—triggered by the experimental treatments or spontaneously along social interactions.

6.3 Within-group dynamics

Beyond treatment assignment, participants' behavior was shaped by emergent dynamics within their groups. Most communities followed a similar engagement pattern, with activity peaking mid-study and declining afterward (see Fig. S5). However, one group developed a distinct norm of upvoting new posts over time—interpreted in post-survey comments as a way to signal acknowledgment. These findings suggest that user experiences reflect not only institutional design but also locally emergent group behavior, which can amplify or reshape the effects of experimental interventions and should be an explicit focus of future research. See also Table S2 for the number of views, comments and the level of toxicity by political issue.

To explore predictors of discussion drop-out over time, we applied survival analysis using a Cox proportional hazards model. This model incorporated treatment condition and group toxicity as predictors of early drop-out (see Fig. S2 A and B). Results indicated that both experimental treatments served as protective factors, reducing the risk of early withdrawal from participation. Conversely, higher levels of group toxicity, as measured by the Jigsaw Perspective API, were associated with an increased risk of early drop-out. These findings further highlight the role of group dynamics in sustaining engagement in discussions.

We also explored predictors for average participant-level comment *scores* (see Fig. S7). Compared to our core analysis of predictors for participation, we find an entirely different pattern: on average, higher scores (via upvotes of comments) are received by women, those with left leaning political orientation (which is the group majority), those who perceive to align with issue attitudes in the group and who do not perceive the group as polarized.

6.4 Statistical Power

The pre-registration did not include ex ante power analyses as our sample size was constrained by budgetary considerations, which limited our ability to plan for the ideal level of statistical power in advance. However, to better understand what our study can and cannot teach us, we have conducted post-hoc power simulations using `simr` with comments as unit of analysis (see Fig. S9). These analyses show that with our realized sample size, the study is well-powered (>80%) to detect, for example, moderate treatment effects on toxicity (such as a 10% change in Toxicity levels) but underpowered for smaller effects (e.g. 5% change in Toxicity) when considering random effects specifications (including random slopes on the level of the subreddit).

Given the subreddit-level dependencies within the structure of our experimental design—participants interacting in groups, clustering standard errors on the level of the subreddit would also be a plausible and more conservative model specification (see Fig. S18). However, with only two subreddits per experimental condition, clustered SEs are extremely fragile, likely overestimating uncertainty, and the design severely underpowered. A random-effects specification, by contrast, allows us to account for subreddit-level dependence while still making use of the large number of individual observations. This approach also better reflects the data-generating process, in which both treatment and subreddit context shape responses, and it supports inference beyond the specific subreddits in the study.

When considering political outcome variables such as political cynicism (we ran power simulations on this exemplary outcome variable as it comes with enough variation to simulate from), we find that with our realized sample size (with political outcomes, the participant is the unit of analysis), we are sufficiently powered to reliably detect effect sizes with beta > 0.3 in the random effects specification (corresponding to a shift of 0.15 SD in the raw (unscaled) outcome). However, the design is underpowered to detect smaller effects—effects that are commonly reported in the literature. We recommend that future research uses our data as guidance when estimating statistical power prior to fielding similar experiments.

6.5 Overjustification under the incentives condition

Regarding interaction activity, when comparing degree distributions of user interaction networks that could also reflect whether users merely "do their job" and directly reply to each post (in the form of root comments), which would be consistent with the literature on overjustification, we do not find significant differences in centrality distributions between experimental conditions. Also descriptively, there are not substantially more "isolated" users who never interact directly with other users when posting their comment (see Figs. S14, S12 and S13). However, there was less commenting on weekends, when no new discussions were opened by the researchers (see Fig. S15).

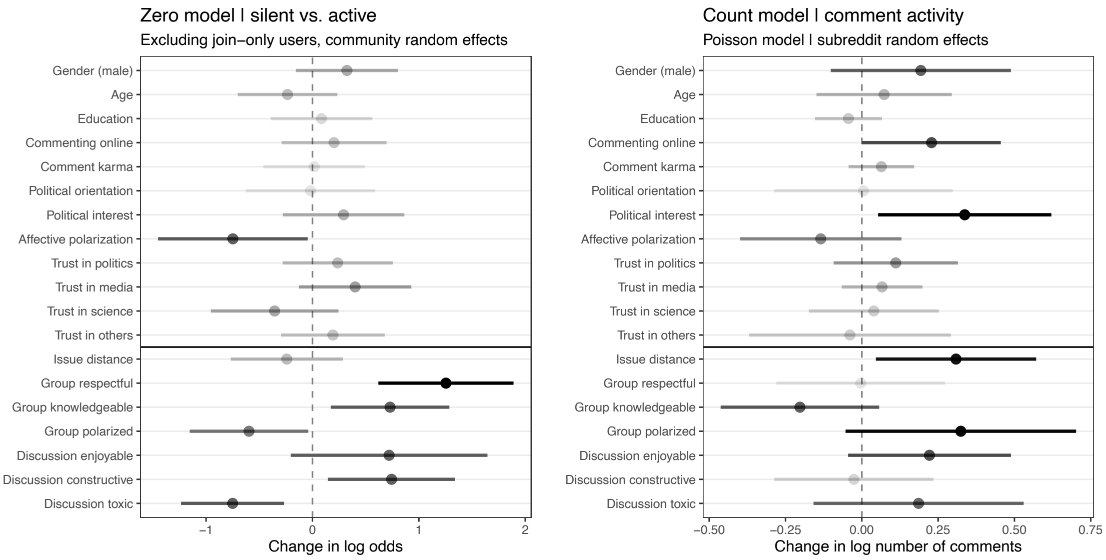

Figure S1: **Models supplementary to analysis of participation predictors (RQ1).** Variant of hurdle model that includes community (subreddit) random effects. This variant serves as robustness check to the analysis reported in Fig. 2 of the main text as with our study design, we are facing dependencies of observations within subreddits—participants interacting in groups. However, as we are not estimating any treatment effect here, we commit to the more simple model in the main text but report this more complex model for full transparency. Hurdle model components were estimated separately with `lme4::glmer()`.

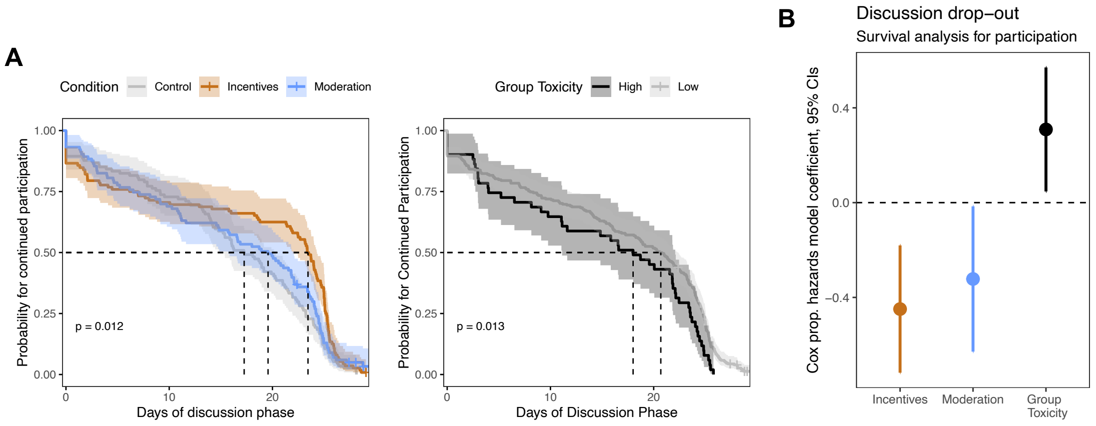

Figure S2: **Discussion drop-out over time.** A: Kaplan–Meier survival curves illustrating the proportion of participants who remained engaged in discussion over time, providing a non-parametric visualization of attrition dynamics. B: Results of a Cox proportional hazards model estimating predictors of discussion drop-out, expressed as hazard ratios. Both experimental treatments demonstrated protective effects, lowering the likelihood of early disengagement relative to the control condition. In contrast, higher levels of group toxicity, as measured by the Perspective API, were significantly associated with an increased hazard of early drop-out, suggesting that toxic conversational environments accelerate participant attrition.

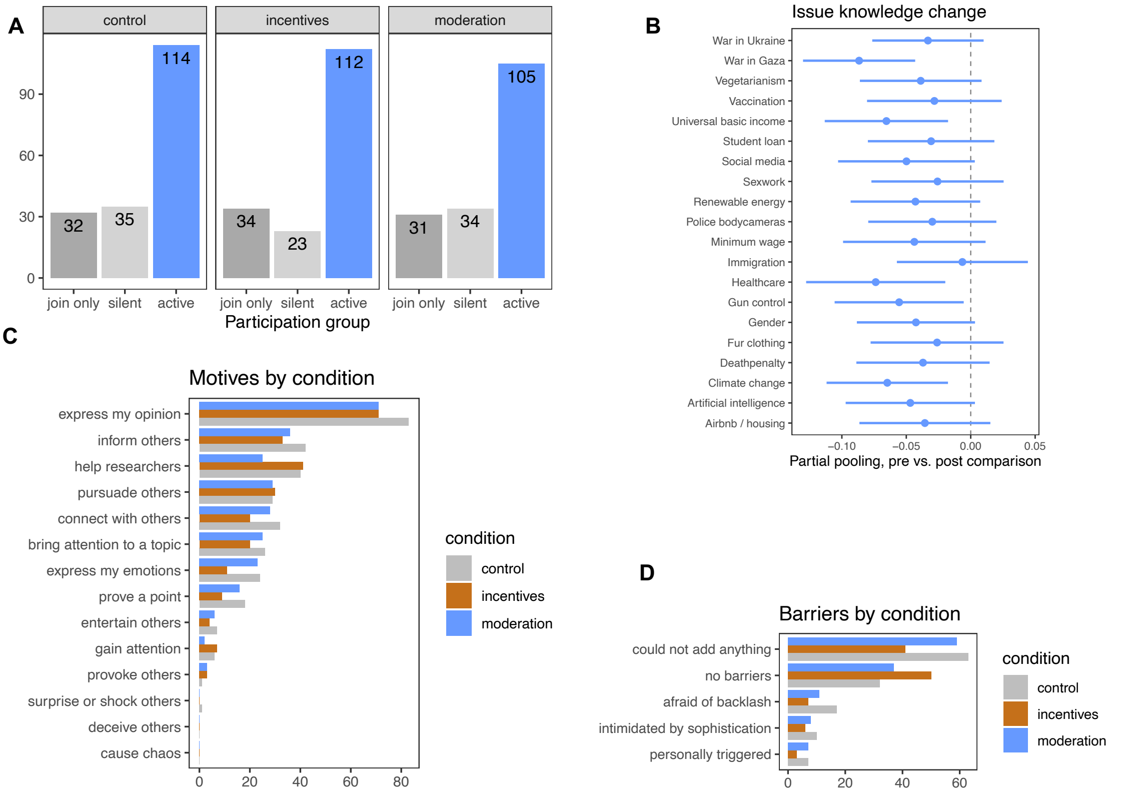

Figure S3: **Supplementary descriptive figures.** A: Participation groups by experimental condition. Reduction of silent users only visible in under the incentives condition (see also Fig. 3 in the main manuscript). B: Descriptive issue knowledge change pre vs. post discussion period. Coefficients represent time coefficients of mixed models with partial pooling: `lmer(knowledge_issue_i ~ time + (1 | ParticipantID) + ( 1 | subreddit)`. We consistently observed lower levels of reported issue knowledge for almost all of the 20 political issues discussed. However, these time effects are purely descriptive and potentially subject to considerable confounding by external political events. This pattern mirrors the finding of reduced political interested, presented in Fig. 6B of the main manuscript. C: Motives expressed in check-in surveys for commenting, split by experimental condition. D: Barriers to commenting by condition.

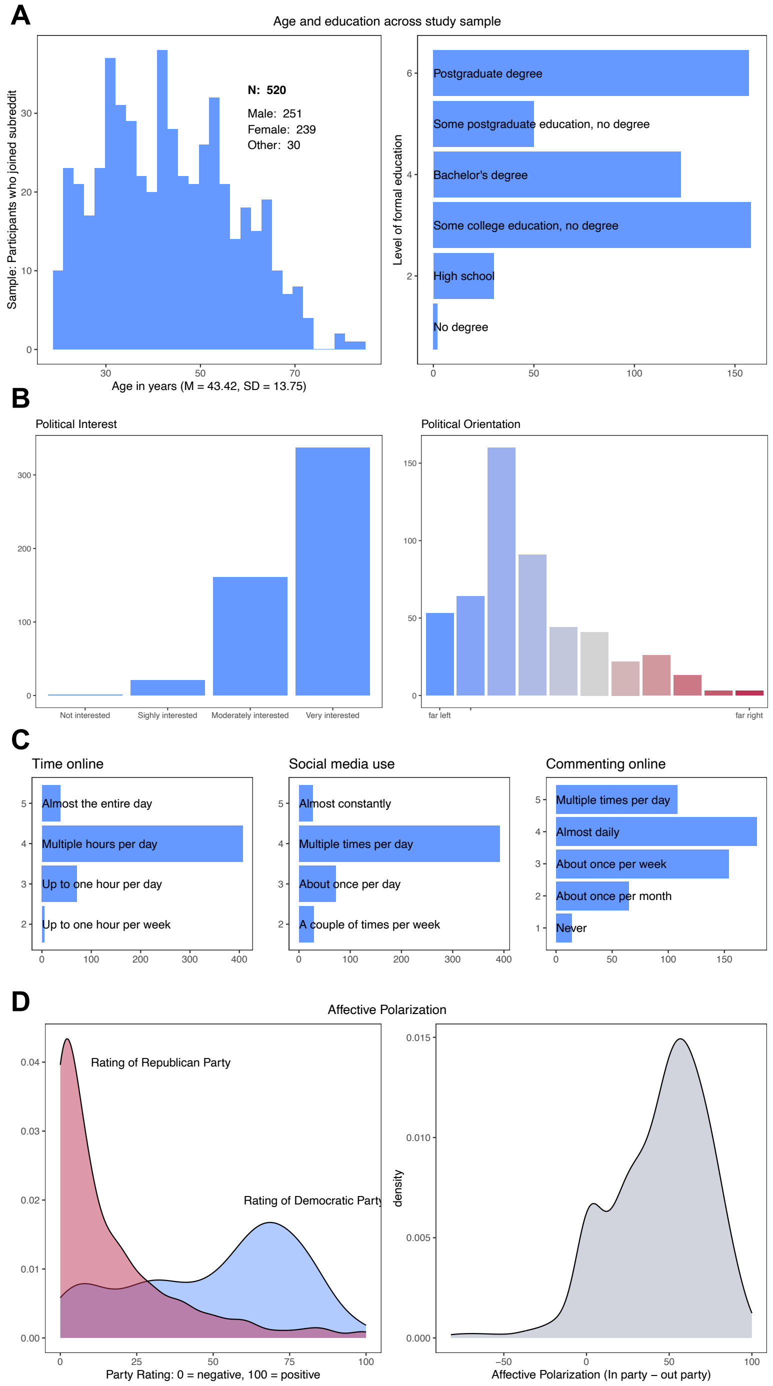

Figure S4: **Demographic information of sample who completed pre-discussion survey and was admitted to discussion communities.** A: Distributions of age, gender and levels of formal education. B: Political interest and political orientation on 10-point left-right scale as reported in pre-treatment survey. C: Self-reported online activity (time spent online, social media use, commenting online). D: Party ratings and affective polarization (in-party - out-party).

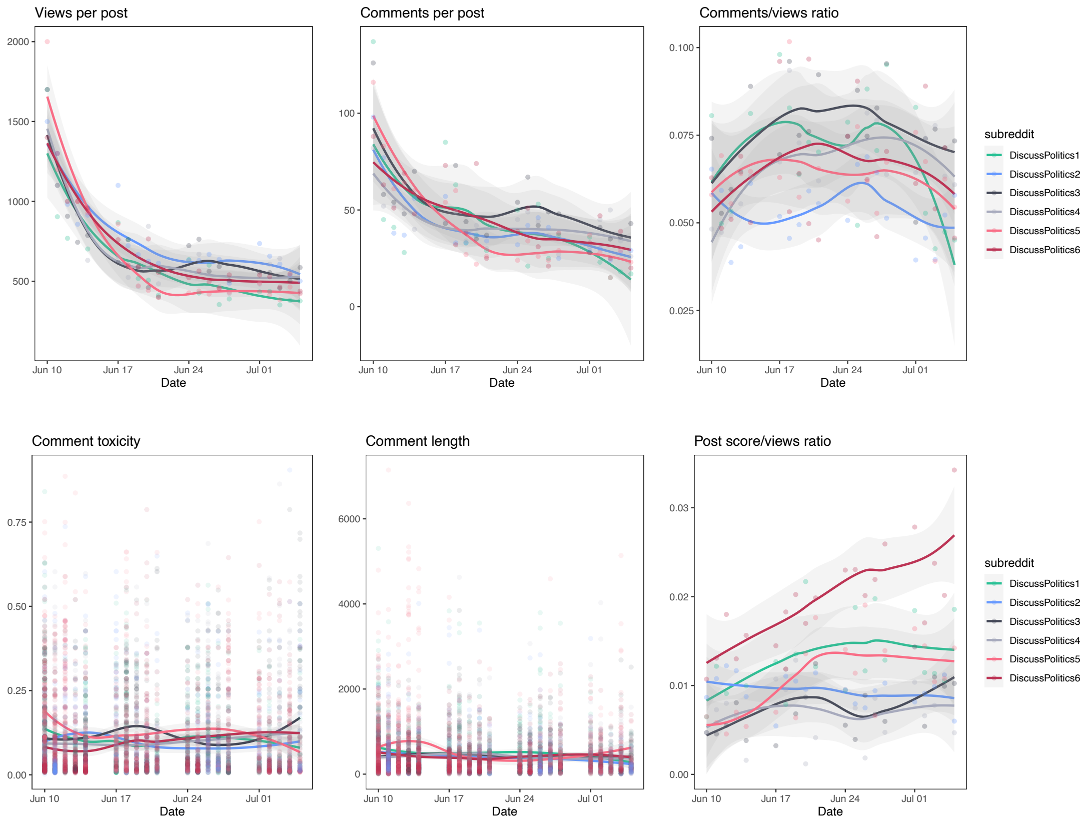

Figure S5: **Reddit metrics over the course of the study.** Top: Engagement metrics aggregated to the discussion thread level. From left: views, comments and comment/view ratio over time, split by community. While, when regarded in isolation, views and comments accumulate on older posts over time; the comments/view ratio peaks around the middle of the discussion phase. Bottom: Comment characteristics over time. From left: toxicity, length. Bottom right: score/view ratio aggregated to discussion thread level. We can see an informal "community norm" emerging with participants in the subreddit "DiscussPolitics6" overproportionally upvoting posts. From free text responses, we learned that some participants used upvoting to demonstrate that they have noted a new post.

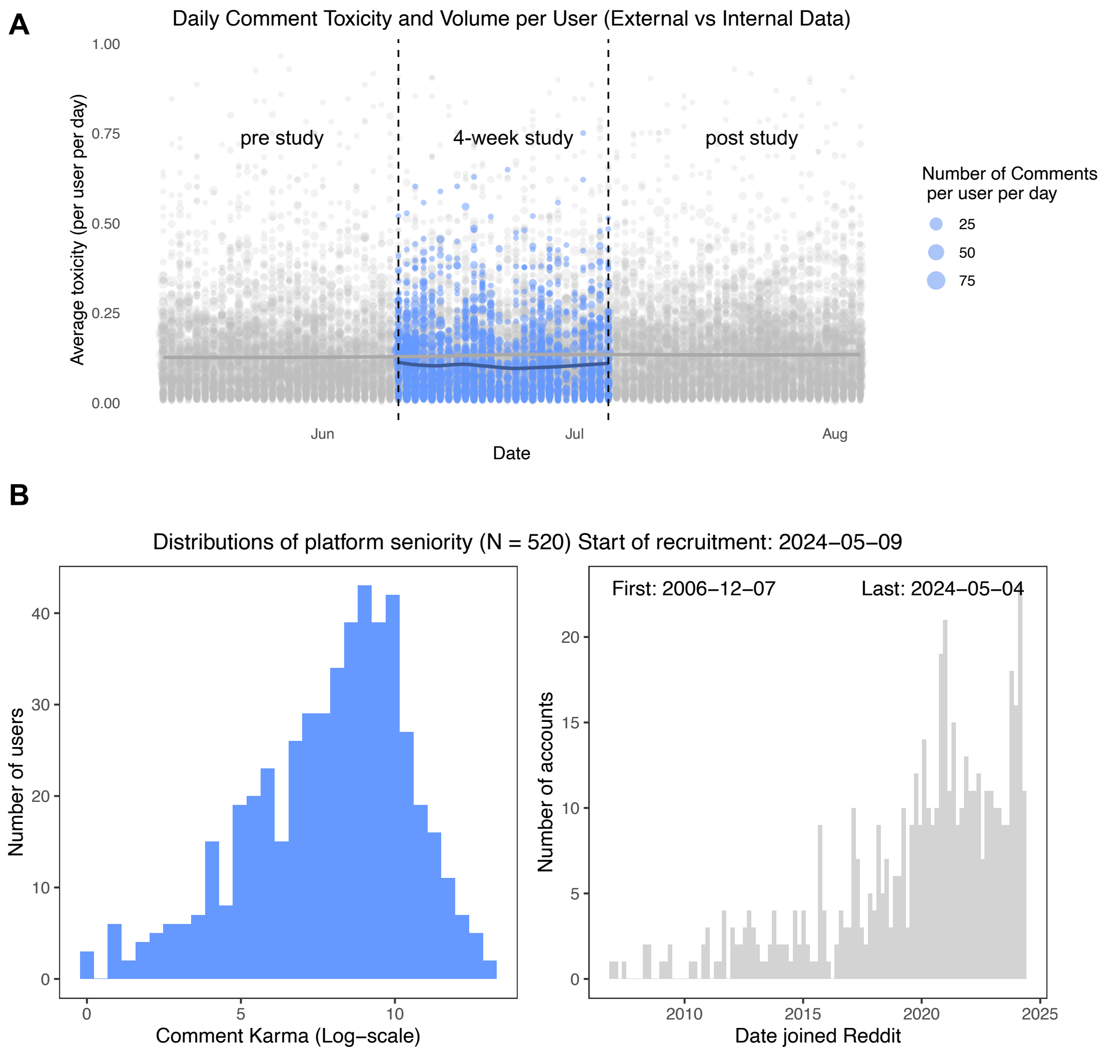

Figure S6: **Supplementary information regarding Reddit metrics.** A: External vs. internal comments collected from participants under informed consent. We see a slightly decreased toxicity level in comments collected throughout the experiment (see also Fig. 5C in the main manuscript). B: User information collected from Reddit. Left: Distribution of comment karma values, used as a proxy for prior commenting activity and platform engagement. Right: Account creation dates (with a cut-off prior to the start of recruitment), providing context on participants' tenure on Reddit and potential variability in platform familiarity.

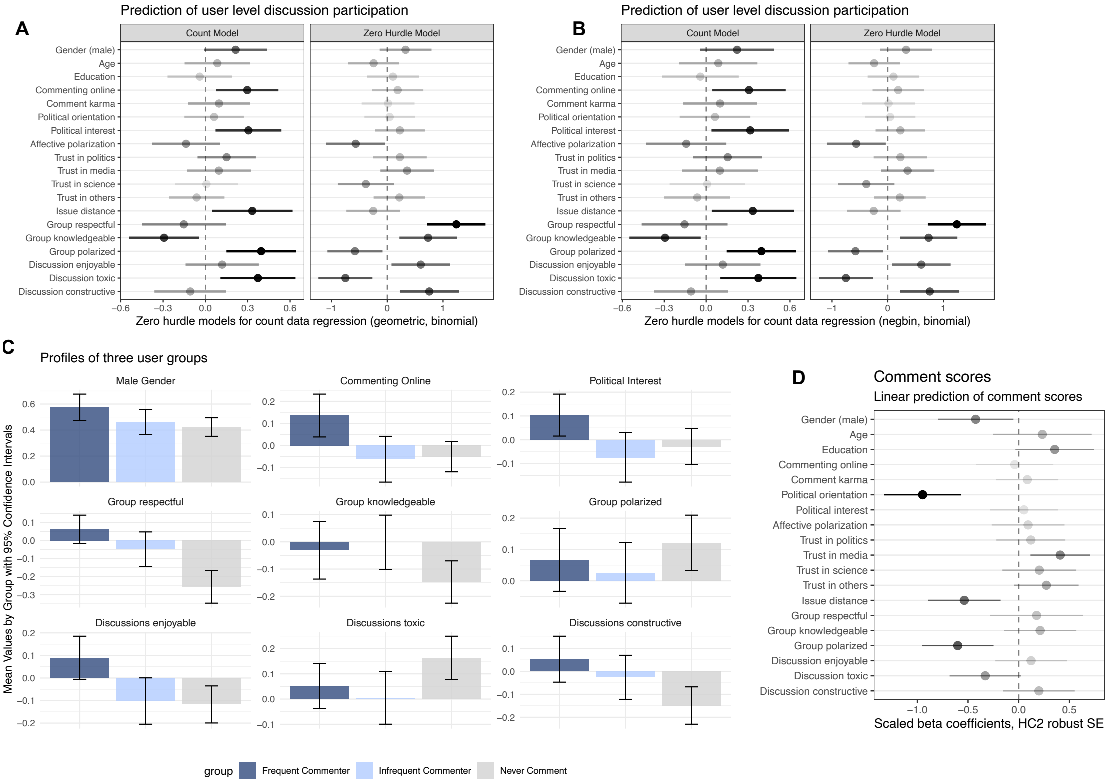

Figure S7: **Models supplementary to the analysis of participation predictors (RQ1).** A: Variant of main analysis with geometric distribution (instead of poisson). B: Variant of main analysis with negative binomial distribution (instead of poisson). These alternative model specifications can be used to accommodate overdispersion in the count data. Zero hurdle models remain unchanged, error bounds widen in the count models but do not change the interpretation of the main results. C: Visual representation of marginal means for different user groups represented in hurdle models. Cut-off for frequent commenter: $> 70\%$ quantile, cut-off for infrequent commenter: $< 30\%$ quantile. Visual representation chosen to provide a more intuitive interpretation of main results. The selected arbitrary cut-offs play no role in main models (see Fig. 2B in the main manuscript). D: User-level prediction of average scores received on comments in experiment (indicating received feedback instead of participation).

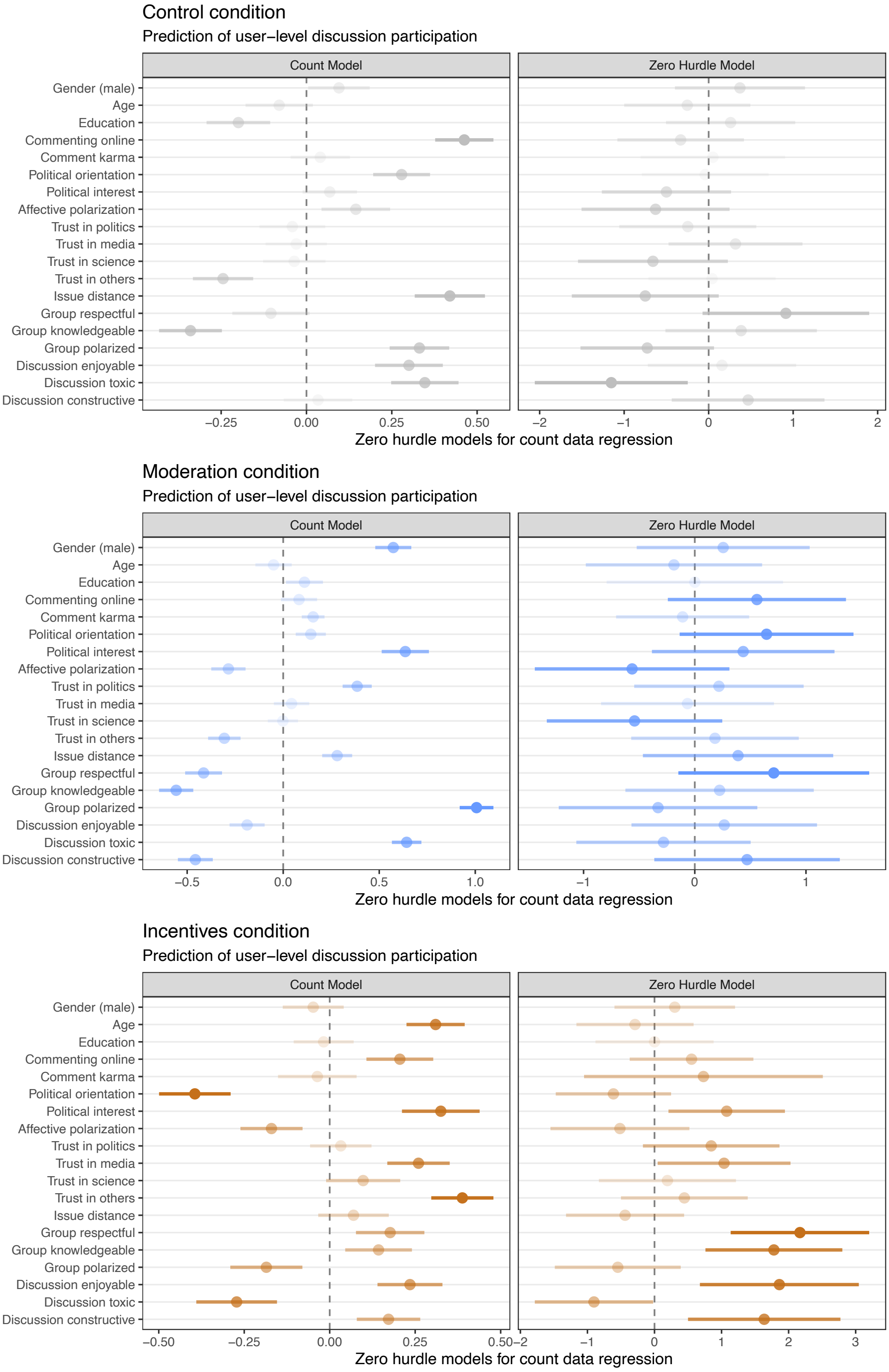

Figure S8: **Zero hurdle models for participation (as in Fig. 2B of the main manuscript), broken down by experimental condition.**

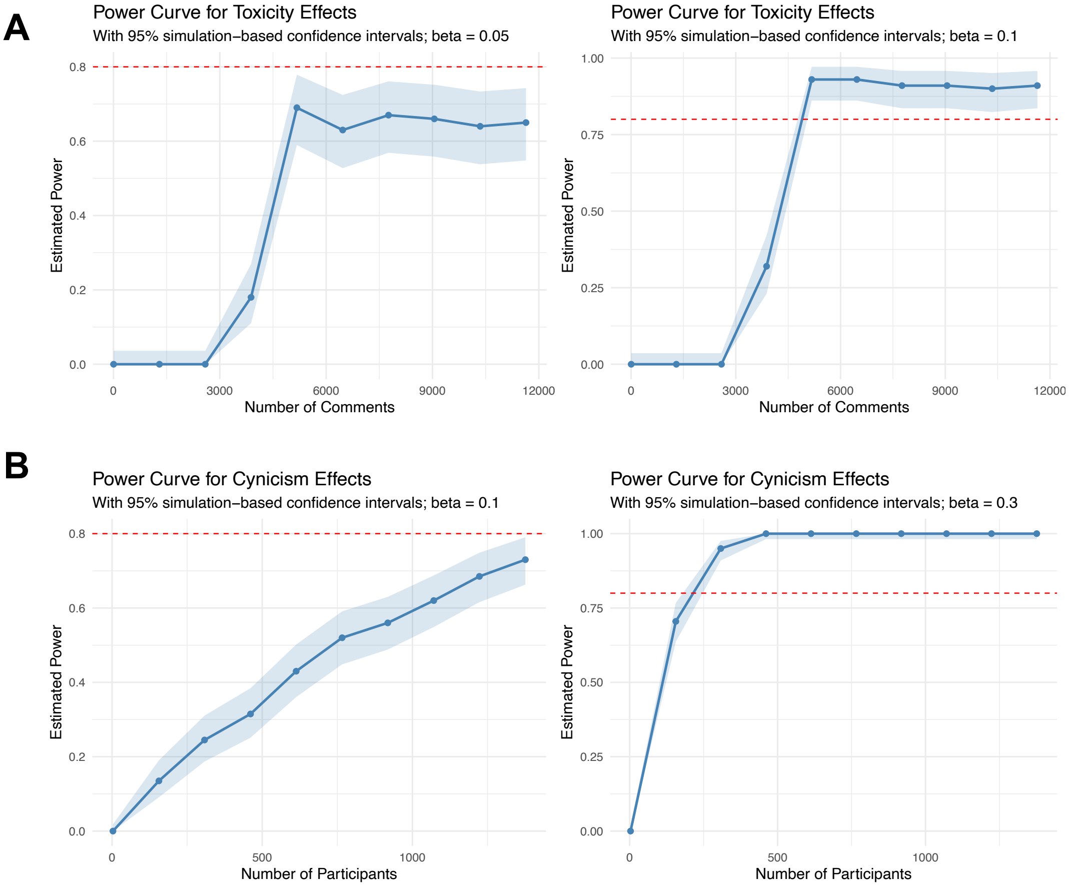

Figure S9: **Power simulations.** (A) Power simulations for toxicity effects (see Fig. 5A in the main manuscript) with random effects specification. `lme4::lmer(toxicity ∼ condition + (1 + condition | subreddit))`. (B) Power simulations for effects on political cynicism (as example political outcome variable) with random effects specification (see Fig. 6A in the main manuscript). Unit of analysis is the participant. `lme4::lmer(cynicism ∼ time * condition + (1 + condition | subreddit) + (1 | ParticipantID))`.

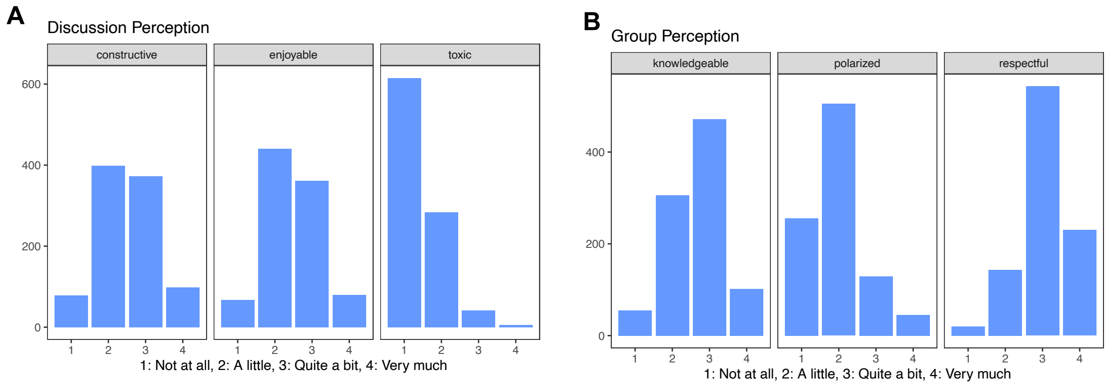

Figure S10: **Summary of responses to questions fielded in check-in surveys.** Regarding discussion (A) and group perceptions (B). Association of these variables with participation reported in Fig. 2B of the main manuscript.

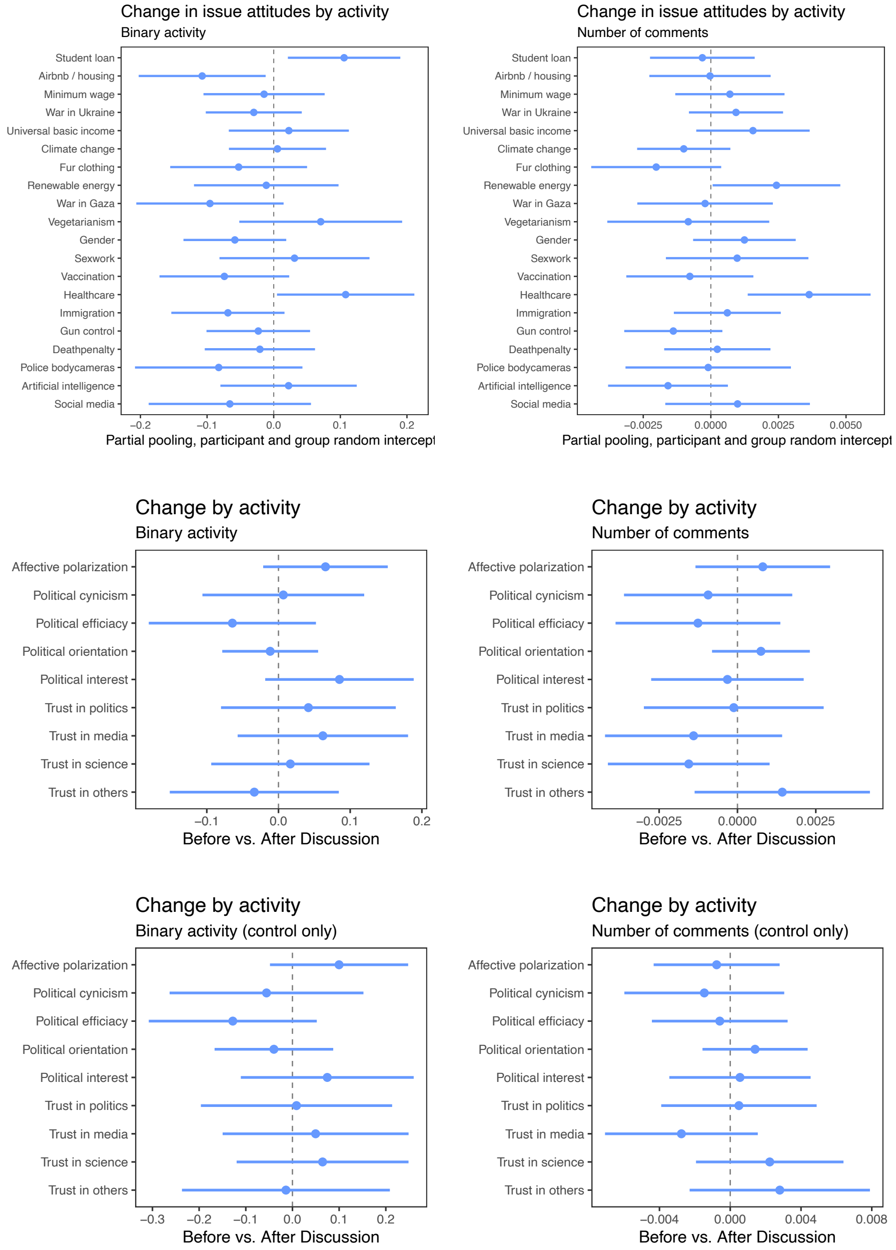

Figure S11: **Supplementary models of change in lurkers vs. power users.** Models examine whether patterns of attitudinal or political change during the study period varied as a function of participants' engagement levels. The left column reports estimates from the zero-hurdle portion of the model (see Fig. 2B of the main manuscript), comparing silent "lurkers" who contributed no comments with active participants who engaged in discussion. The right column reports estimates from the count model (also corresponding to main Fig. 2B), contrasting participants with high versus low commenting activity ("power users" vs. lower-activity participants). Across both specifications, results indicate minimal differences in pre- to post-discussion changes in issue-specific attitudes or broader political variables, suggesting that discussion activity levels were not a major driver of attitudinal change.

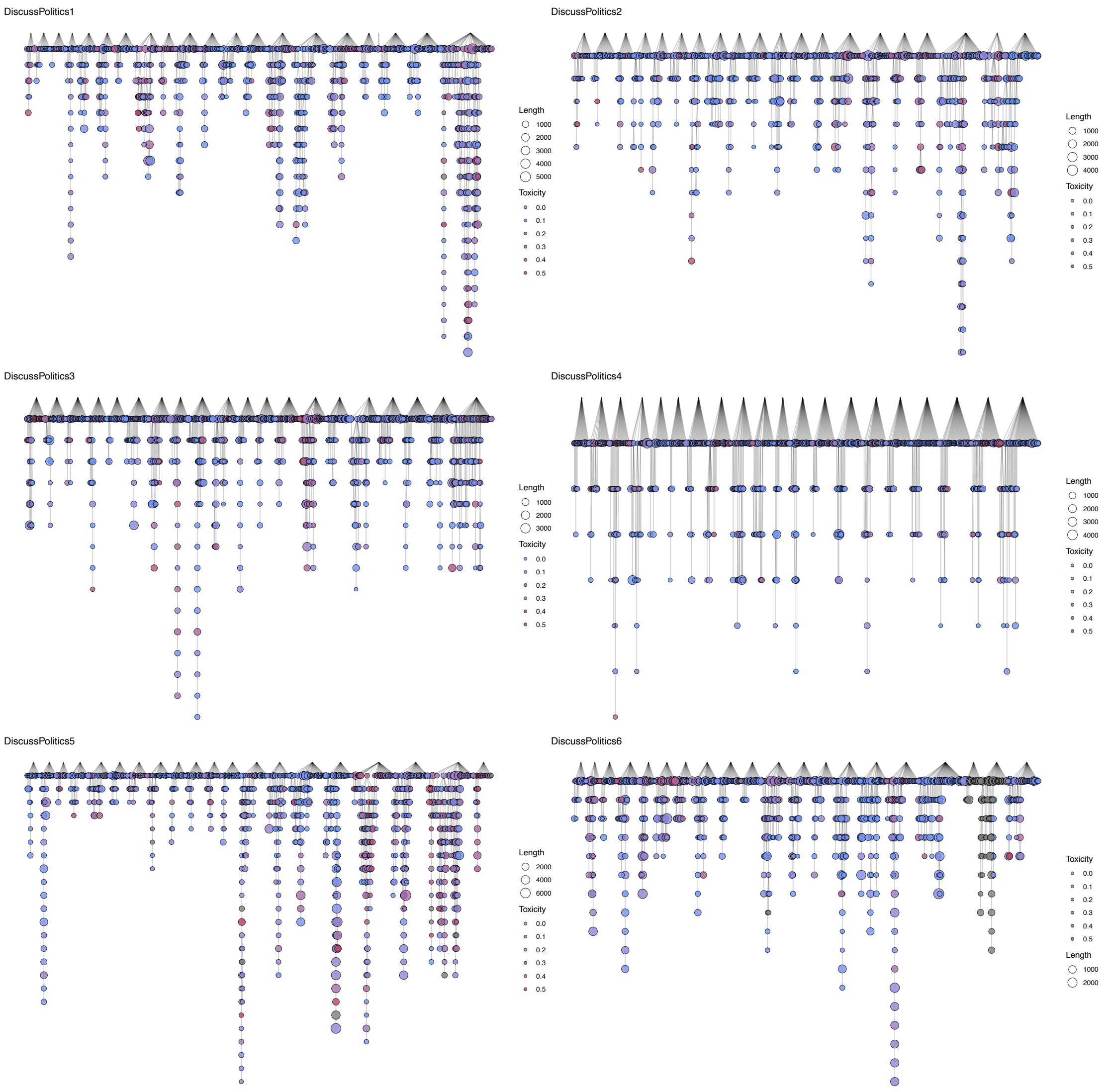

Figure S12: **Discussion threads displayed as trees.** Visualization of the discussion data, shown here as illustrative tree structures. The top row depicts the 20 political issue seed posts created by researchers to initiate discussions, with participant comments branching below each seed post. Thread depth and branching patterns illustrate how conversations unfolded, providing a structural overview of discussion dynamics rather than detailed content.

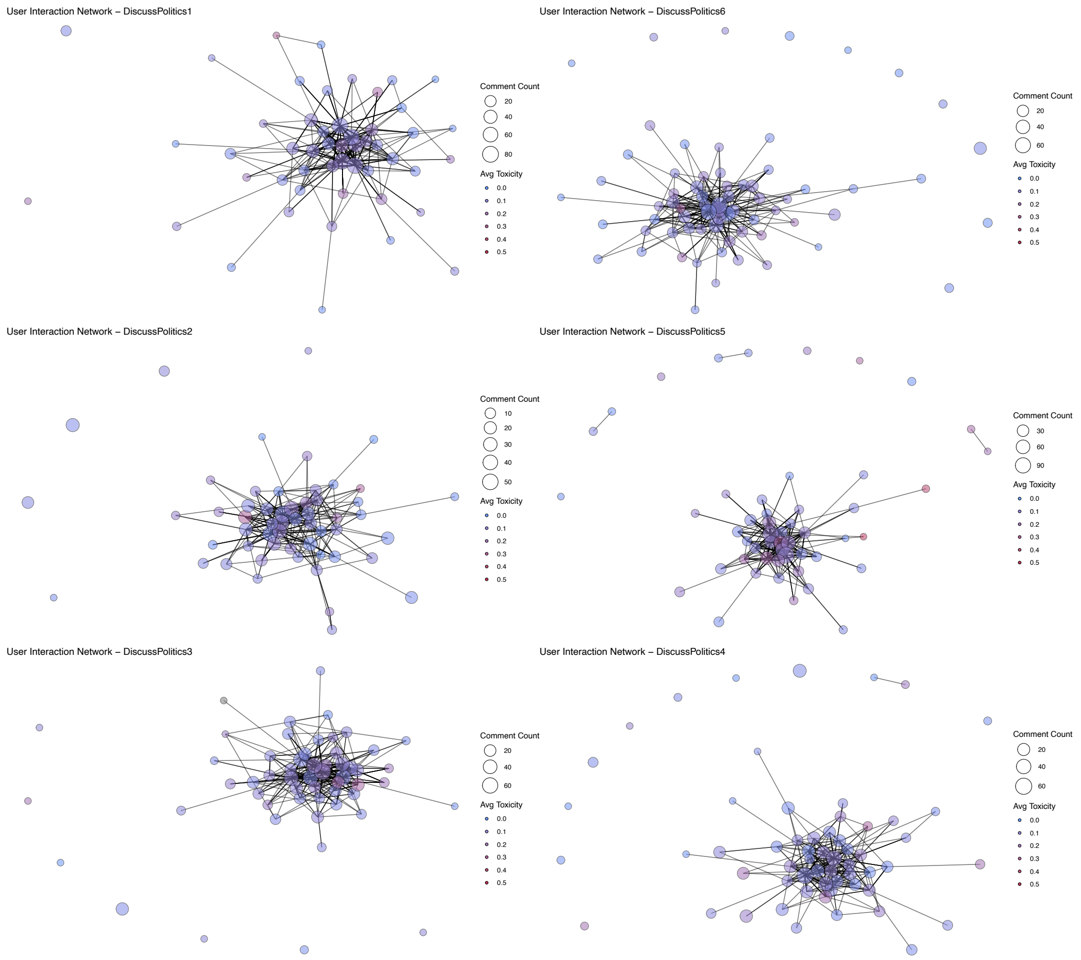

Figure S13: **User interaction networks for each community.** Visualization of participant interaction networks, with edges indicating instances where one user replied directly to another user's comment. Isolated nodes represent users who only commented on the original researcher seed posts without engaging in replies to others. Descriptively, the incentives condition does not appear to generate more isolated users, suggesting no evidence of overjustification effects (i.e., participants merely fulfilling paid requirements to comment once per day). Instead, interaction patterns remain comparable across conditions, indicating that incentives did not undermine motivation to participate in discussions.

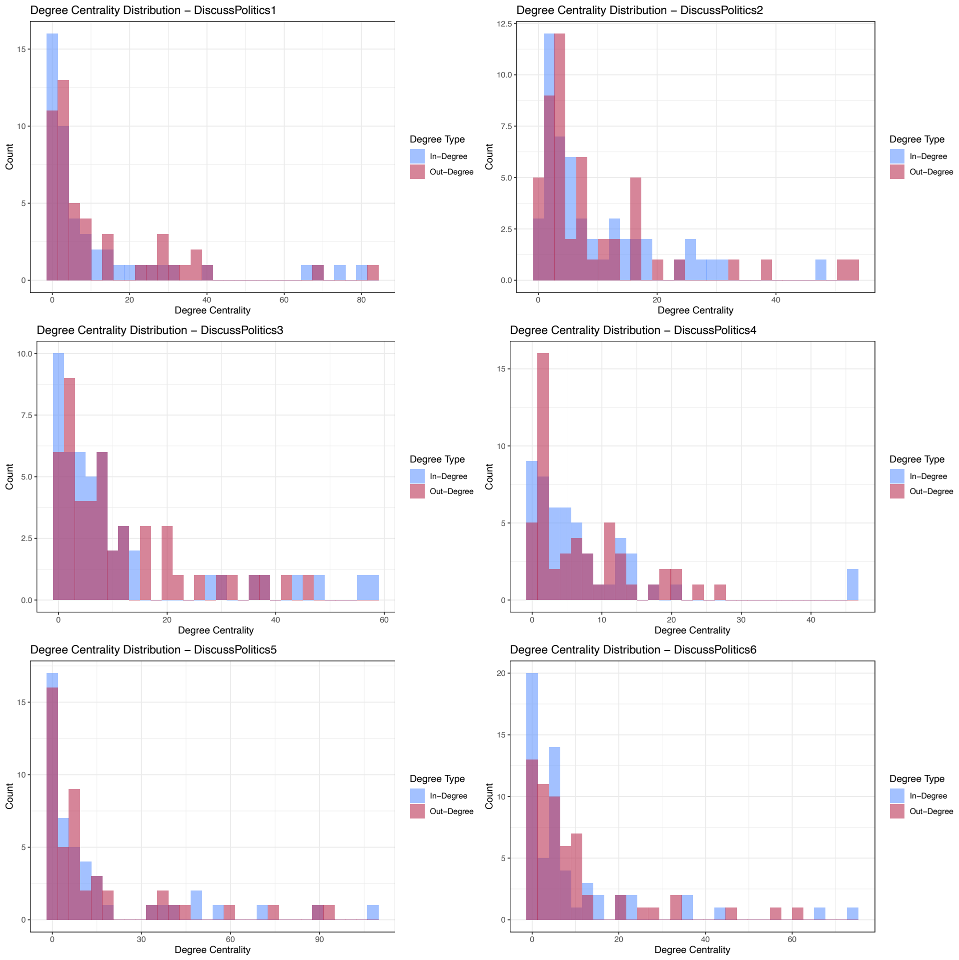

Pairwise Kolmogorov-Smirnov Test

Comparison	D	p	Centrality
Control vs. moderation	0.094	0.76	In-degree
Control vs. incentives	0.100	0.68	In-degree
Control vs. moderation	0.054	0.99	Out-degree
Control vs. incentives	0.090	0.81	Out-degree

Note. Degree centrality distributions from user interaction networks in communities. Edge generation if user replies to other user (not seed post).

Figure S14: **Characteristics of user interaction networks.** Statistical analysis of interaction structures across discussion communities. Top: Degree distributions of user networks, with edges defined by one participant replying to another participant's comment (excluding replies to the original researcher seed posts). These distributions capture the extent to which users engaged with one another and the variability in number of connections formed. Bottom: Results of Kolmogorov–Smirnov tests comparing degree distributions between experimental conditions. Consistent with the descriptive network visualizations, statistical tests reveal no significant differences across conditions, indicating that experimental treatments did not alter the overall connectivity patterns of participant interactions.

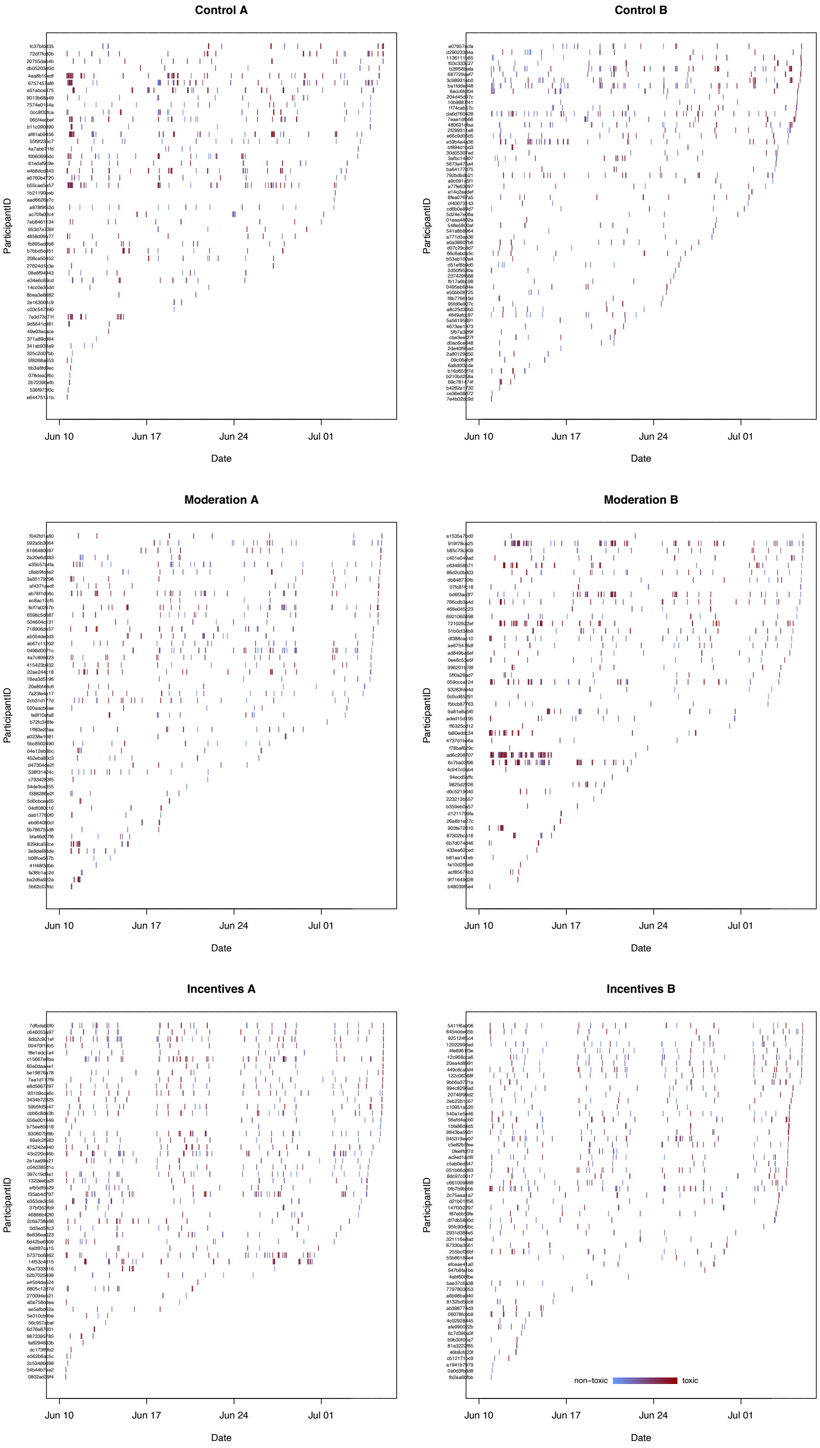

Figure S15: **Raw Participation Plot.** Coloured by Toxicity; each rectangle represents one comment, each sub-plot represents one community. Participants within communities are sorted by the timing of their last comment, showing that around half of (active) participants write comments until the end of the discussion phase while the times of last comments of the remaining half distributes evenly across the discussion phase with no temporal clusters in drop-out. Descriptively, participants in the incentives condition show slighly more "clean" weekends with no commenting activity. Moderation B shows one participant with a cluster of toxic comments within the first week of the discussion period—a pattern that may explain wider standard errors in the models of comment toxicity by condition (see Fig. 5A in the main manuscript).

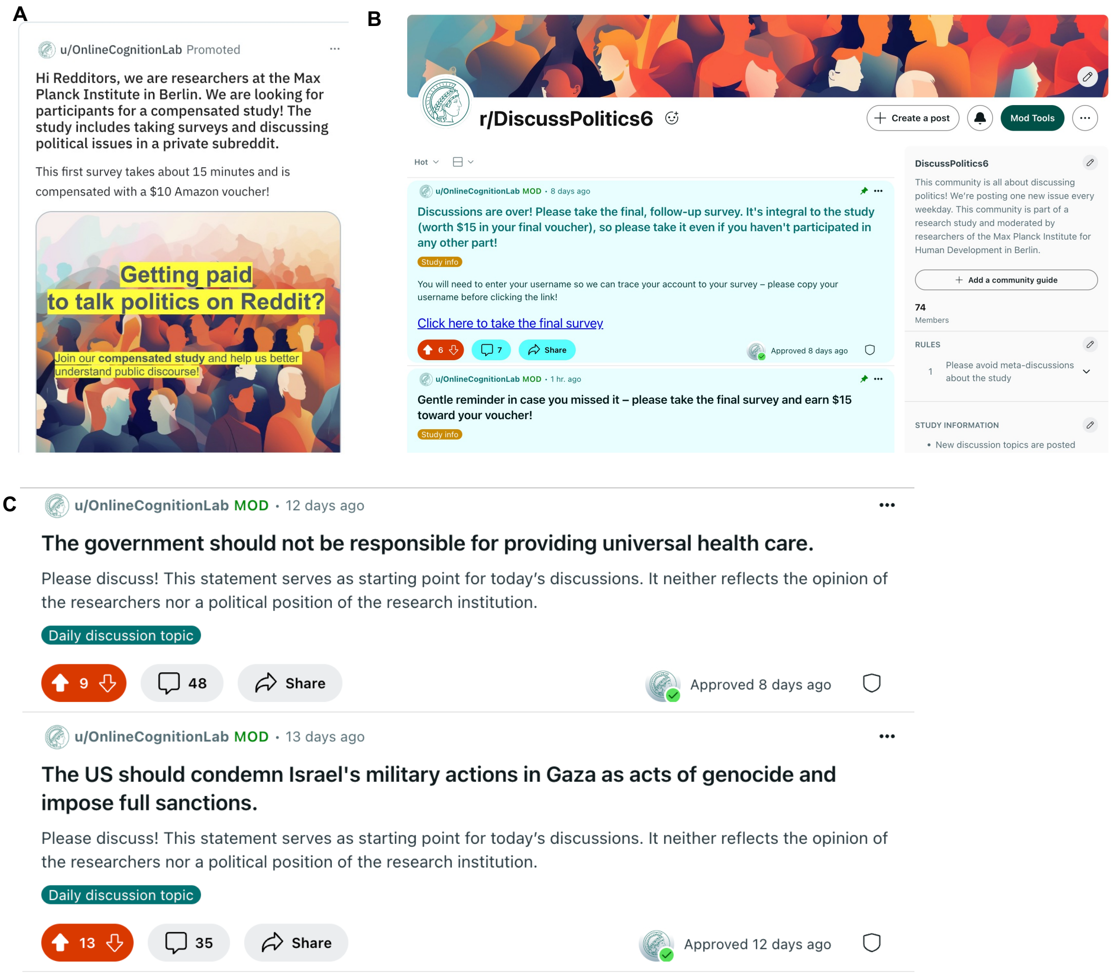

Figure S16: **Study materials visible to participants.** A: Reddit advertisement used for participant recruitment, B: Discussion community (subreddit) user interface visible to participants, C: example seed posts as visible to participants during discussion phase.

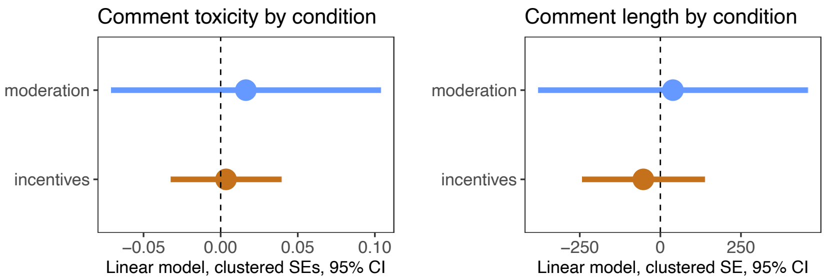

Figure S17: **Treatment effects with standard errors clustered on the subreddit level.** Most conservative model specification of the analysis presented in Fig. 5A of the main manuscript. Model specification: `estimatr::lm_robust(toxicity ~ condition, clusters = subreddit)`.

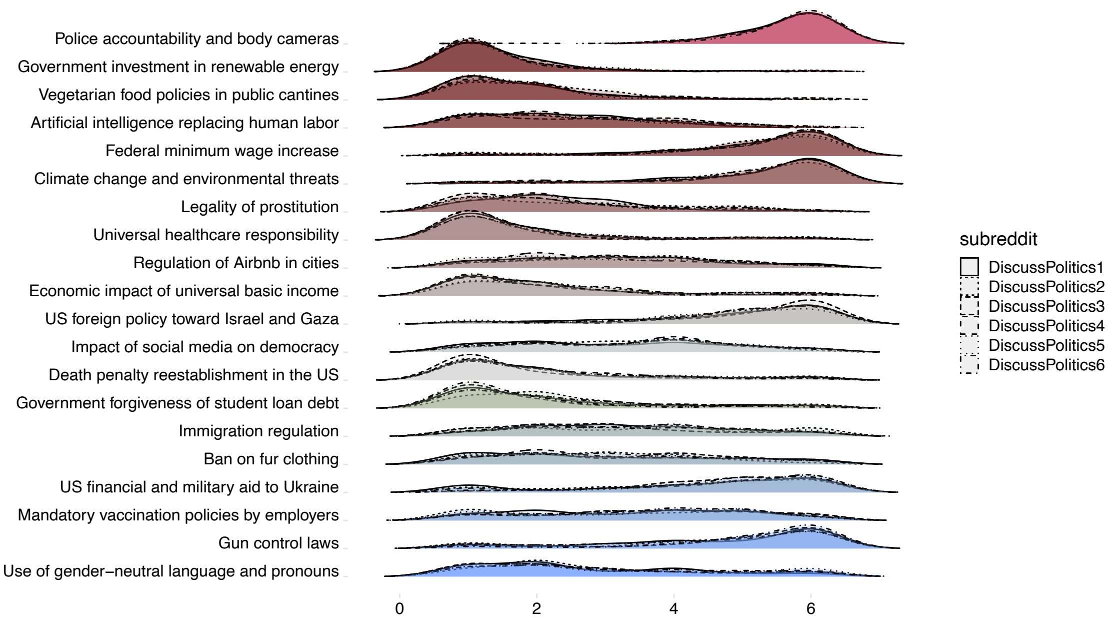

Figure S18: **Pre-treatment issue attitudes.** Distributions of self-reported pre-treatment issue attitudes across different subreddits (scale ranging from 1: strongly disagree to 6: strongly agree, note that some statements were phrased negatively). Issues ranked and coloured by standard deviation with issues displayed at the top showing smallest standard deviations across the entire dataset. While some issues show more variance in self-reported opinions than others, there is no bimodal distribution on any issue.

Table S1: Individual-Level Predictors of Attrition After Subreddit Admission

**Predictor**	**Estimate**	**SE**	z	p
Condition: incentives	0.12	0.29	0.40	.686
Condition: moderation	0.13	0.30	0.45	.649
Age	0.39	0.26	1.46	.144
Gender (male)	-0.27	0.25	-1.06	.290
Education	-0.46	0.25	-1.83	.068
**Comments online (W1)**	-0.50	0.25	-1.97	**.048**
Comment karma	-0.05	0.29	-0.18	.854
**Left–Right (W1)**	-0.59	0.29	-2.03	**.042**
Political interest (W1)	-0.18	0.26	-0.67	.501
Affective polarization (W1)	-0.27	0.26	-1.03	.302

Note. Binomial model predicting "join-only" status (did not answer any surveys beyond the pre-treatment survey and did not write any comments in discussion phase after being admitted to subreddits) vs. lurker or active user status.

Table S2: Discussion seed posts with metrics

Issue	Toxicity	Comments	Views
The US should condemn Israel's military actions in Gaza as acts of genocide and impose full sanctions.	0.191	435	5535
Prostitution should be illegal.	0.190	213	3134
Things like gender-neutral language and stating pronouns are silly issues.	0.183	538	3926
The death penalty should be reestablished US-wide.	0.149	388	4985
The US should provide financial and military aid to Ukraine.	0.136	263	3892
We need stricter gun control laws.	0.118	338	4472
Social media is a threat to democracy.	0.108	233	3460
Immigration should be regulated more strictly.	0.101	286	4235
Fur clothing should be banned.	0.096	230	3311
Police officers should wear body cameras.	0.092	313	3854
Climate change is one of the greatest threats to humanity.	0.084	223	2863
Employers should mandate vaccination.	0.073	258	3183
The government should not be responsible for providing universal health care.	0.073	220	3326
The government should not forgive student loan debt.	0.069	296	3850
Artificial intelligence should replace humans where possible.	0.068	293	4832
There should only be vegetarian food in cantines.	0.065	290	4683
A universal basic income would kill the economy.	0.064	225	3185
The federal minimum wage should be increased.	0.063	305	4321
The government should not invest in renewable energy.	0.060	198	3576
Airbnb should be banned in cities.	0.059	274	3043

Table S3: Baseline Balance (Demographics, Political Variables)

	control	incentives	moderation	p	SMD
n	181	169	170		
Age	43.46 (13.90)	43.03 (13.69)	43.77 (13.71)	0.883	0.036
Gender	1.62 (0.59)	1.54 (0.61)	1.56 (0.60)	0.442	0.089
Education	4.28 (1.35)	4.25 (1.38)	4.27 (1.31)	0.982	0.013
Time online	3.93 (0.50)	3.89 (0.44)	3.92 (0.53)	0.743	0.056
Social media use	3.77 (0.64)	3.81 (0.60)	3.83 (0.60)	0.628	0.066
Writing comments	3.48 (1.08)	3.59 (1.00)	3.68 (1.02)	0.189	0.130
Political orientation (left–right)	3.73 (2.06)	3.85 (2.04)	4.16 (2.18)	0.150	0.134
Political interest	3.60 (0.62)	3.60 (0.56)	3.61 (0.55)	0.971	0.017
Rating Democrats	53.20 (25.74)	50.31 (27.37)	48.66 (27.65)	0.278	0.113
Rating Republicans	16.51 (21.50)	15.16 (21.11)	16.46 (19.67)	0.795	0.043
Trust politics	1.77 (0.62)	1.84 (0.63)	1.81 (0.69)	0.629	0.069
Trust media	2.08 (0.67)	2.08 (0.68)	2.06 (0.66)	0.941	0.024
Trust science	3.62 (0.55)	3.63 (0.53)	3.49 (0.68)	0.058	0.152
Trust others	2.40 (0.72)	2.46 (0.66)	2.32 (0.73)	0.195	0.131
Political cynicism	4.17 (0.99)	3.99 (1.05)	4.16 (1.08)	0.204	0.114
Affective polarization	46.36 (28.59)	45.01 (28.05)	41.25 (30.20)	0.267	0.117
Political efficacy	3.03 (0.35)	3.01 (0.30)	3.03 (0.35)	0.865	0.036
Comment Karma	17451.63 (42386.03)	16819.56 (36107.33)	25077.38 (65773.47)	0.233	0.103
External comment count	136.05 (169.42)	159.14 (164.32)	165.42 (176.26)	0.291	0.115
External comment mean score	6.22 (6.61)	7.64 (7.23)	8.63 (11.69)	0.059	0.187
External comment mean toxicity	0.12 (0.07)	0.13 (0.07)	0.12 (0.06)	0.672	0.071
External comment mean length	229.17 (173.18)	205.92 (136.93)	192.37 (124.69)	0.093	0.165

Note: Values are displayed as means and standard deviations (SDs). SMD = Standardized mean difference.

Table S4: Baseline Balance (Political Issue Attitude and Knowledge)

	control	incentives	moderation	p	SMD
Attitude student loans	1.96 (1.43)	2.20 (1.55)	2.41 (1.58)	0.020	0.201
Attitude Airbnb	3.12 (1.46)	3.15 (1.44)	3.08 (1.49)	0.904	0.033
Attitude minimum wage	5.24 (1.19)	5.20 (1.28)	4.87 (1.51)	0.019	0.179
Attitude Ukraine	4.49 (1.61)	4.61 (1.48)	4.36 (1.70)	0.354	0.105
Attitude universal basic income	1.98 (1.33)	2.21 (1.50)	2.45 (1.53)	0.012	0.213
Attitude climate change	5.19 (1.24)	5.02 (1.50)	5.02 (1.42)	0.425	0.084
Attitude fur	3.08 (1.62)	3.18 (1.57)	2.91 (1.48)	0.254	0.120
Attitude renewable energy	1.41 (0.93)	1.43 (0.90)	1.61 (1.20)	0.158	0.120
Attitude Gaza	4.81 (1.44)	4.92 (1.45)	4.68 (1.51)	0.320	0.109
Attitude vegetarianism	1.75 (0.97)	1.89 (1.16)	1.83 (1.07)	0.458	0.089
Attitude gender	2.74 (1.63)	2.92 (1.70)	3.06 (1.73)	0.208	0.126
Attitude prostitution	2.40 (1.37)	2.25 (1.30)	2.49 (1.50)	0.282	0.115
Attitude vaccines	3.53 (1.55)	3.62 (1.60)	3.35 (1.68)	0.279	0.113
Attitude healthcare	1.69 (1.29)	1.85 (1.44)	2.03 (1.51)	0.075	0.163
Attitude immigration	3.31 (1.50)	3.21 (1.51)	3.48 (1.65)	0.260	0.117
Knowledge student loans	3.05 (0.81)	3.11 (0.78)	3.08 (0.80)	0.764	0.052
Knowledge Airbnb	2.53 (0.76)	2.64 (0.92)	2.58 (0.79)	0.425	0.092
Knowledge minimum wage	3.18 (0.68)	3.20 (0.72)	3.11 (0.73)	0.439	0.089
Knowledge Ukraine	2.86 (0.79)	3.01 (0.75)	3.02 (0.74)	0.086	0.140
Knowledge universal basic income	2.70 (0.75)	2.85 (0.71)	2.74 (0.76)	0.126	0.142
Knowledge climate change	3.24 (0.73)	3.33 (0.71)	3.30 (0.65)	0.440	0.089
Knowledge fur	2.31 (0.80)	2.28 (0.84)	2.29 (0.94)	0.962	0.020
Knowledge renewable energy	2.94 (0.72)	3.07 (0.72)	2.98 (0.77)	0.261	0.115
Knowledge Middle East	2.82 (0.72)	2.95 (0.74)	2.85 (0.75)	0.257	0.112
Knowledge vegetarianism	2.76 (0.79)	2.79 (0.94)	2.81 (0.85)	0.857	0.039

Note: Variable means and standard deviations (SDs). SMD = Standardized mean difference.
